# Consensus Forecasting of Species Distributions: The Effects of Niche Model Performance and Niche Properties

**DOI:** 10.1371/journal.pone.0120056

**Published:** 2015-03-18

**Authors:** Lei Zhang, Shirong Liu, Pengsen Sun, Tongli Wang, Guangyu Wang, Xudong Zhang, Linlin Wang

**Affiliations:** 1 Research Institute of Forestry, Chinese Academy of Forestry, Key Laboratory of Forest Silviculture of the State Forestry Administration, Beijing, China; 2 Institute of Forest Ecology, Environment and Protection, Chinese Academy of Forestry, Beijing, China; 3 Centre for Forest Conservation Genetics, Department of Forest Sciences, University of British Columbia, Vancouver, British Columbia, Canada; 4 Faculty of Forestry, University of British Columbia, Vancouver, British Columbia, Canada; 5 Beijing University of Agriculture, Beijing, China; Trier University, GERMANY

## Abstract

Ensemble forecasting is advocated as a way of reducing uncertainty in species distribution modeling (SDM). This is because it is expected to balance accuracy and robustness of SDM models. However, there are little available data regarding the spatial similarity of the combined distribution maps generated by different consensus approaches. Here, using eight niche-based models, nine split-sample calibration bouts (or nine random model-training subsets), and nine climate change scenarios, the distributions of 32 forest tree species in China were simulated under current and future climate conditions. The forecasting ensembles were combined to determine final consensual prediction maps for target species using three simple consensus approaches (average, frequency, and median [PCA]). Species’ geographic ranges changed (area change and shifting distance) in response to climate change, but the three consensual projections did not differ significantly with respect to how much or in which direction, but they did differ with respect to the spatial similarity of the three consensual predictions. Incongruent areas were observed primarily at the edges of species’ ranges. Multiple stepwise regression models showed the three factors (niche marginality and specialization, and niche model accuracy) to be related to the observed variations in consensual prediction maps among consensus approaches. Spatial correspondence among prediction maps was the highest when niche model accuracy was high and marginality and specialization were low. The difference in spatial predictions suggested that more attention should be paid to the range of spatial uncertainty before any decisions regarding specialist species can be made based on map outputs. The niche properties and single-model predictive performance provide promising insights that may further understanding of uncertainties in SDM.

## Introduction

Species distribution modeling (SDM) techniques, which attempt to provide detailed predictions of distributions by statistically relating present-day species distribution to environmental predictors, have been widely used to model and project the changes of species’ geographic distributions in response to climate change[1]. The most important criticism of niche models is their failure to take biotic interactions, evolutionary change, and dispersal processes into account [2,3]. Many of the biological processes are not easily predictable under current and future environmental conditions at either the continental or regional scale [3]. Niche-based models postulate that species distribution and environmental conditions are in a state of equilibrium. Specifically, the speed of plant migration is consistent with that of climate change. Unfortunately, there is often a time lag between changes in environmental conditions and species migration into a more suitable habitat from a newly unsuitable one [4,5]. Correlative (not causal) models have shown considerable predictive accuracy in current distribution simulations, but not all of them have high model transferability [6–8]. Due to the limited availability of biological processes (e.g. species dispersal processes), only simulated data regarding potentially suitable habitats have been generated. These were created based on the environmental conditions of these species’ existing niches. In this way, niche models are only an incomplete description of the relationships between species distribution and environment. Despite conceptual and technical shortcomings, niche-based static models are still considered a suitable first approximation of climate-change-induced effects on species geographical distribution at a large scale because of their simplicity and flexibility when used for a large number of species [1,2,9]. Process-based models that can make predictions of species range shifts at the continental scale are still rare, but they are not yet widely used because they require life histories and physiologies of each species [1, 2, 9]. The amount of information required for each species limits process-based models to only a small number of species [1,9].

In practice, any modeling exercise into an unknown future involves uncertainty. Four sources of uncertainty in niche-based SDM have been identified, including initial dataset conditions (IC), model classes (MC), model parameters (MP), and boundary conditions (BC) [10–12]. IC refers to an incomplete realization of species distribution (e.g. sample size [13,14], range size [15]). Projected changes in species range may differ substantially in both magnitude and direction due to the use of alternate MCs [2,11,16–18]. Predictions are also subject to MP selection. For instance, the two user-defined parameters of random forest (RF) (the number of trees and the number of randomly selected variables to split the nodes) should be optimized to improve predictive accuracy [19]. BC affects distribution projection because of future variations in climate caused by different global circulation models (GCM) and Special Report of Emission Scenarios (SRES) outcomes [15, 20–22].

Uncertainty in distribution projections can skew policy making and planning intended to address and respond to climate change, especially where the conservation of threatened and endangered species is concerned [23]. One recent recommendation is to fit a number of alternative models and to explore the range of projections across more than one set of IC, MP, MC, and BC combinations (herein termed ensemble forecasting) and then to find consensus in model projections (herein termed consensus forecasting) [7,10,24]. The accuracy of the forecast can be substantially improved by combining multiple individual forecasts [25]. Ensemble forecasting has been applied to a variety of fields [10]. Applications involving ensemble forecasting in SDM are still in their infancy, and little is known about the relative performance of different consensus approaches in handling and combining large groups of projections [7,24]. Previous studies on the use of consensual approaches have focused primarily on assessment of model-level variations and have not taken the differences among GCMs into account. However, climate models may involve much less uncertainty than statistical methods in the prediction of species distribution [12,21,22]. The majority of these assessments have focused on the accuracy of prediction as a measure of the performance of consensus approaches [7,24]; few studies have quantified the spatial similarity among consensual prediction maps generated by different consensus approaches. Consensual prediction maps are expected to see increasing use for decision-making in resource management or designation of land in conservation planning. In this way, measuring the incongruent area among consensual predictions may provide important information about the adequacy of consensual approaches that may not be apparent from global comparison in terms of prediction accuracy.

Most available studies have demonstrated that species distribution traits could substantially influence the accuracy of species distribution predictions [15,20,21]. To date, there is no single niche model that always provides the most accurate predictions for all species [6,26]. A general conclusion is that habitat-specialist species yield models are more accurate than habitat-generalist ones [16,26]. However, the manner in which different traits of species distribution patterns affect the results of the consensual predictions derived from different consensus approaches remains unexplored. For consensus approaches to be used effectively in biodiversity and conservation management, thorough examinations of their relevance to species with different geographical distribution characteristics are necessary.

In this study, using eight niche models, nine random data-splitting bouts and nine different climate change scenarios, the distributions of 32 forest tree species in China were simulated under current and projected future climate conditions. Forecasting ensembles were combined by means of three widely-used consensus approaches, i.e., on the basis of median (PCA), average, and frequency of species occurrence under given climatic conditions. The primary objectives were 1) to determine whether there is substantial variation in consensual prediction maps among different consensus approaches and 2) to determine whether these variations could be best explained by species traits and niche mode predictive performance.

## Materials and Methods

### Study area and plant species

The study area encompassed all of China. Thirty-two common forest tree species, which collectively account for more than 50% of forest cover in China, were selected for comparison of various consensus approaches in projecting the species distribution under current and future climate and potential range shifts. See S1 Table for ecological requirements and biological characteristics of these 32 tree species. Information regarding the current distribution of the 32 tree species was originally derived from the Vegetation Distribution Map of China (1:1,000,000 scale)[27]. They were then rasterized to a cell size of 8 km×8 km. The data consisted solely of whether any individual of these 32 tree species was present in the area. These data, together with a soil map of China (see below), were obtained from the Environmental and Ecological Science Data Center for West China of the National Natural Science Foundation of China (http://westdc.westgis.ac.cn).

### Environmental variables

Seven climatically derived variables are considered critical to plant physiological function and survival: mean annual temperature (MAT,°C), mean warmest month temperature (MWMT,°C), mean coldest month temperature (MCMT,°C), difference (TD,°C) between MWMT and MCMT, mean annual precipitation (mm), mean annual summer precipitation (May to September, mm), and degree-days above 5°C (DD,°C). Baseline climate data were averaged for the period 1961–1990. These seven climatic variables were calculated using ClimateChina (Ver 4.4) [28], which was developed using the same methodologies as ClimateBC [29]. See S1 Appendix for detailed information on ClimateChina. In addition to climatic variables, 10 soil variables known to affect plant species distributions were also selected: organic matter content (%), N, P, K content (%), coarse, fine, silty, clay sand content (%), soil depth (cm), and pH. These soil variables were derived from the 1:1,000,000 scale soil map of China database and rescaled to a spatial resolution of 8 km × 8 km to match the species data grid. A total of 17 environmental predictor variables were selected based on understanding of their biological relevance to the distribution of plant species.

### Future climate scenarios

To assess the uncertainties related to future projections of climate change, three SRES emissions scenarios (A2, A1B, and B1) and three GCMs (MIROC32_medres, Center for Climate System Research at the University of Tokyo, National Institute for Environmental Studies, and Frontier Research Center for Global Change; CCCMA_CGCM3, Canadian Centre for Climate Modeling and Analysis; BCCR-BCM2.0, Bjerknes Centre for Climate Research) were used in this study. Climate change scenarios were averaged for three 30-year periods: 2010–2039 (2020s), 2040–2069 (2050s), and 2070–2099 (2080s). For the future climatic projections, the same set of seven climate variables were calculated using ClimateChina software for all 8 km×8 km grids.

### Niche models

The distribution of the 32 tree species was predicted using a BIOMOD framework [30] programmed in R software [31]. BIOMOD includes eight niche-based models: generalized linear models (GLM), generalized additive models (GAM), multivariate adaptive regression spines (MARS), mixture discriminant analysis (MDA), classification tree analysis (CTA), generalized boosting method (GBM), artificial neural network (ANN), and RF. MPs were selected based on modeling techniques. One set of MPs was assigned to each model for each split-sample bout. For example, RF and GBM needed the maximum number of trees to be specified. We used three target degrees of freedom for smoothing spline in the GAM [15,30].

### Pseudo-absence selection and split-sample

All eight niche-based models require species presence and absence records. One solution was to generate pseudo-absences when no reliable absence data were available [13,32,33]. Recent studies have indicated that pseudo-absence data should be restricted to locations where conditions are distinctly unsuitable for this species occurrence [13,34]. To improve sampling accuracy, method described by Engler et al. (2004) [34], was used to select absences with a presence-only environment envelop model (surface response envelop model, SRE). SRE is a submodel of the BIOMOD platform, which identifies locations where all predictor variables fall within the extreme values (both maximum and minimum limits of each predictor) as determined by species occurrence sites. Any site identified by SRE was precluded from pseudo-absences, and the remaining pseudo-absences were considered true absences.

Then 70% true absences were selected for model development. This may prevent bias attributable to inclusion of an extremely high number of absences and reduce the computation burden [32,35]. The prediction dataset (i.e. 70% true absences plus entire presence) was randomly divided into a set of calibration data and a set of testing data at a ratio of 7:3. In order to have an equal chance of selecting true absences and splitting data, these two processes were replicated three times each (i.e. nine random training and testing subsets were generated) to reduce variability in model-building process and subsequent predictions. In the data splitting process, the ratio between the number of presences and absences in the calibration and testing dataset was kept to be constant. In this way, a total of 72 different models were calibrated for each species.

### Model evaluation

The evaluation dataset generated by split-sample was used to assess the accuracy of the model. Model accuracy was determined using three measures: The Kappa, true skill statistic (TSS), and area under the curve values (AUC) of receiver operator characteristic (ROC) curves. These three measures attribute different weights to the various types of prediction errors (e.g. omission, commission or confusion). AUC is an effective, threshold-independent model evaluation indicator and is also independent of prevalence (i.e. the frequency of occurrence) of target species [36]. AUC values below 0.7 were here considered poor, 0.7–0.9 moderate, and > 0.9 good. Both Kappa and TSS are threshold-dependent measures of model accuracy. They both ranged from −1 to +1, where +1 indicates perfect agreement between predictions and observations and values of 0 or less indicate agreement no better than random classification [37]. The following ranges were used to interpret Kappa and TSS statistics: values < 0.4 were poor, 0.4–0.8 useful, and > 0.8 good to excellent.

### Combination of ensemble forecasting

A total of 648 projections (72 models × 3 GCMs × 3 SERSs) were generated for each tree species. To reduce uncertainty in species distribution projections, the following three most-widely used consensus approaches were used to combine ensemble model projections after deleting models with AUC < 0.70, Kappa, and TSS < 0.4.

#### Median (PCA) approach

A two-step method was used: (1) An individual projection was selected among the eight niche-based model projections for each of the nine split-sample bouts and each of the nine climate scenarios by using principal components analysis (PCA) (e.g. [7,24]). These projections were closely correlated to the PCA consensus axis (or the first principal component) and represented the general trend of model projections (see also [6]). (2) The median values of the 81 projections (nine predictions for current distribution) selected by the first step were then computed to integrate modeling uncertainties and to represent the final consensus forecast of future distributions.

#### Average

The simple average of all models outputs (predictions or projections) was calculated.

#### Frequency

The frequency of predictions or projections that indicated a given species was present in each grid was calculated after transforming each probability map into binary values (presence/absence) at threshold 0.5.

### Statistical analysis

Repeated-measures ANOVA was used to analyze the variations in model performance (AUC) between model classes and species. Model classes and species types served as fixed factors and nine split-sample bouts served as random factors. The analysis was performed using the linear mixed effect model of R statistical package (“lme” function) [38].

Because all consensus approaches produced predictive probabilities, the comparisons of range area changes among different projections require a threshold to classify predicted presences and absences. A species is considered present at a given grid if the probability of occurrence is above 0.5. To track changes in latitudinal distributions, we compared geographic centers (or centroids) of current and future species range. The geographic center for probability value of each species was calculated by using the mean center function in ArcGIS9.3 (ESRI Inc., http://www.esri.com/). The coordinates of the centroid were used to calculate distance and direction of habitat shift. A two-way ANOVA was performed to investigate variations in the changes in species range among three consensus approaches for each time period using species changes in range (i.e. area change percentage or shifting distance) as a response variable and consensus approach and tree species as factors. Because the levels in the factor (tree species), are a sample of possibilities (i.e. other trees), we could think the factors as random effects.

Pearson’s correlation coefficient was used to quantify spatial similarity among prediction maps for each species, pairwise among consensus approaches. Kappa value was also used to evaluate the similarity of prediction maps after the probability maps were transformed into a binary presence-absence map using a 0.5 threshold. To distinctively characterize the incongruent pattern between species distribution maps, the current work focused on the locations where the probability of species occurrence was above 0.5 as predicted by any one of the three consensus approaches.

To investigate which variable best explains spatial correspondence among consensual prediction maps, the average single-model predictive accuracies (AUC, Kappa, and TSS) and six species ecological and biogeographical properties (prevalence, specialization, marginality and latitudinal, thermal, and elevation ranges) were defined as explanatory variables. Pairwise map correlations (Pearson’s correlation and Kappa) were averaged among all consensus approaches for each species for baseline and future time to use as the dependent variables and related differences in map correlation to explanatory variables using multiple stepwise regression models.

The latitudinal and elevation ranges were described as the differences between the average values of the 10% most extreme sites (maximum and minimum) where each species was found. To define the species thermal range, a PCA was performed on the five thermal variables (MAT, MWMT, MCMT, TD, and DD). The first two axes of this PCA, which account for more than 95% of the total variability, were kept as a synthetic variable describing thermal gradients. The thermal range was calculated as the difference between the average positions of the 10% highest and lowest values along this synthetic variable where each species was found. Prevalence is here defined as the proportion of species’ presence in the model-training data. The two measures of environmental niche, specialization and marginality, were calculated using ecological niche factor analyses (ENFA in R package “adehabitatHS” [39]). Specialization describes the species’ niche breadth by comparing variability in environmental conditions within a species’ range to the variability in environmental conditions in the entire study area. Strong specialization indicates that the niche is narrow. Marginality is a measure of the departure between the species optimum and the mean environmental conditions in the study area and is therefore representative of the species’ ecological niche position.

## Results

### Niche model performance

**Fig 1:**
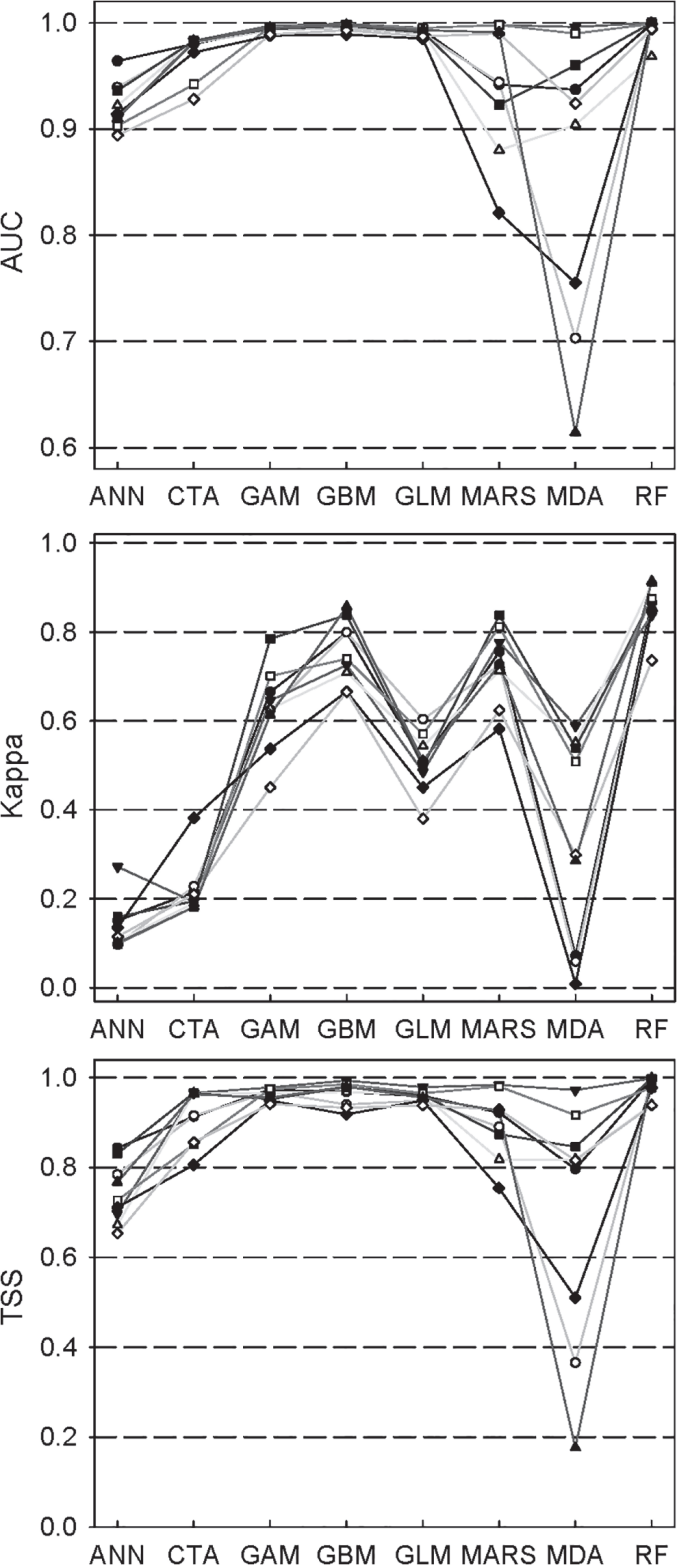
Predictive accuracies (AUC, Kappa, and TSS) of *Larix principis-rupprechtii*. Nine different symbol types (dark circles) indicate nine random split-sample bouts (original data were randomly divided into two sets: a calibration set and a validation set) and the same symbols are linked by the same straight lines.

The split-sample procedure (or data-splitting process) influenced the model performance (Fig. 1; S2 Table). For *Larix principis-rupprechtii*, niche models, like RF, GAM, GBM, and GLM, showed higher predictive accuracy (AUC, Kappa, and TSS) and were less sensitive to the procedure than other models (Fig. 1).

**Fig 2:**
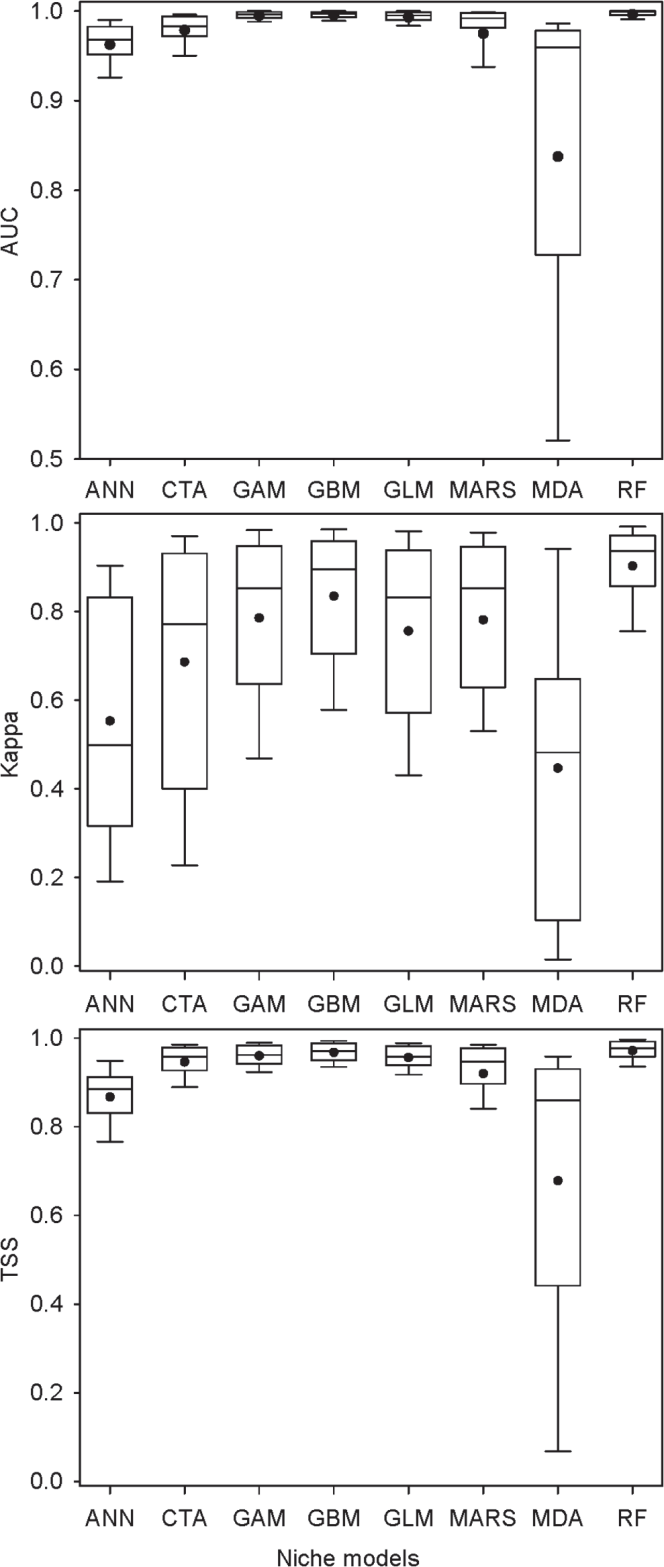
Box-whisker plot of differences in model performance (AUC, Kappa, and TSS) among model classes when data were pooled for all species and split-sample bouts. Dots show the mean predictive accuracy across species and split-sample bouts.

The predictive accuracies of the models also varied among modeling techniques when data were pooled for all species and split-sample bouts, with MDA showing the worst average performance and largest deviation. It was followed by ANN, CTA, and MARS. RF, GLM, GAM, and GBM showed the better average performance (Fig. 2).

Variations in predictive accuracy were clearly demonstrated among the 32 species when data were pooled for all niche models and split-sample bouts (S1 Fig.). These observations were consistent with repeated-measure ANOVA analysis, which indicated the variations in model performance among modeling approaches and tree species were significant (Table 1).

**Table 1:** Repeated-measures ANOVA assessing changes in model predictive accuracy (AUC, Kappa and TSS) between modeling approaches and tree species.

Metrics	Source of variation	Numerator freedom	Denominator freedom	F-value	*P*-value
AUC	Intercept	1	2257	395733.1	<0.0001
	Niche models	7	2257	75.6	<0.0001
	Tree species	31	2257	2.3	0.0001
Kappa	Intercept	1	2257	78272.5	<0.0001
	Niche models	7	2257	197.0	<0.0001
	Tree species	31	2257	85.8	<0.0001
TSS	Intercept	1	2257	142053.9	<0.0001
	Niche models	7	2257	135.9	<0.0001
	Tree species	31	2257	7.4	<0.0001

Linear mixed effects models were used to perform this analysis with niche models (eight levels) and tree species (32 levels) as fixed factors and split-sample replicates (nine levels) as random factors.

A simple regression model was used to evaluate the relationship between average model performance (AUC, Kappa, and TSS) and species traits (latitudinal, thermal, and elevation ranges, prevalence, specialization, and marginality). Results from this analysis showed that AUC and TSS were negatively related to latitudinal and elevation ranges (Table 2). Prevalence was the only significant predictor that was positively correlated with Kappa.

**Table 2:** Linear regression modeling of the effects of specie traits on niche model performance.

		Model parameters		
		Coefficient	*P*-value	R square
AUC	Elevation range	−6.629E-06	0.001	0.296
	Latitudinal range	−0.001	0.021	0.164
Kappa	Prevalence	3.746	0.001	0.314
TSS	Elevation range	−2.688E-05	0.001	0.335
	Latitudinal range	-0.006	0.003	0.261

### Changes in species range

Changes in the potential distribution area of tree species and changes in distance and direction of mean centers of suitable habitat were predicted for the periods of 2020s, 2050s and 2080s using three different consensus forecasting methods. The period 1961–1990 served as a baseline. Results are presented in S3 Table. Of the 32 tree species, 27 were consistently predicted using three consensus approaches to expand their potential distribution ranges (3.7–107.4%) or contract their potential habitats (0.4–86.0%) under altered climate, whereas the remaining five species did not change in concert (S3 Table). In future climates, most tree species showed a consistent tendency to shift their ranges in the same direction (northwest or southwest) according to the three consensus approaches (S3 Table). Two-way ANOVA indicated no significant difference in species’ relative changes in range (changes in relative area and distance of range shift) among three consensus approaches (Table 3).

**Table 3:** Significance (*P*-value) of difference in the changes in species’ range (relative to baseline) predicted by three different consensual approaches.

Time	Change in area			Distance of range shift	
	Total range change	New habitat	Habitat lost	Northward shift	Eastward shift
2020s	0.365	0.072	0.063	0.253	0.513
2050s	0.638	0.263	0.469	0.328	0.308
2080s	0.635	0.568	0.822	0.222	0.345

A two-way ANOVA was used to investigate differences in the changes in species range among three consensual approaches for each time period using changes in species range as the response variable and consensual approach and tree species as factors.

### Comparison of consensual prediction maps

**Fig 3:**
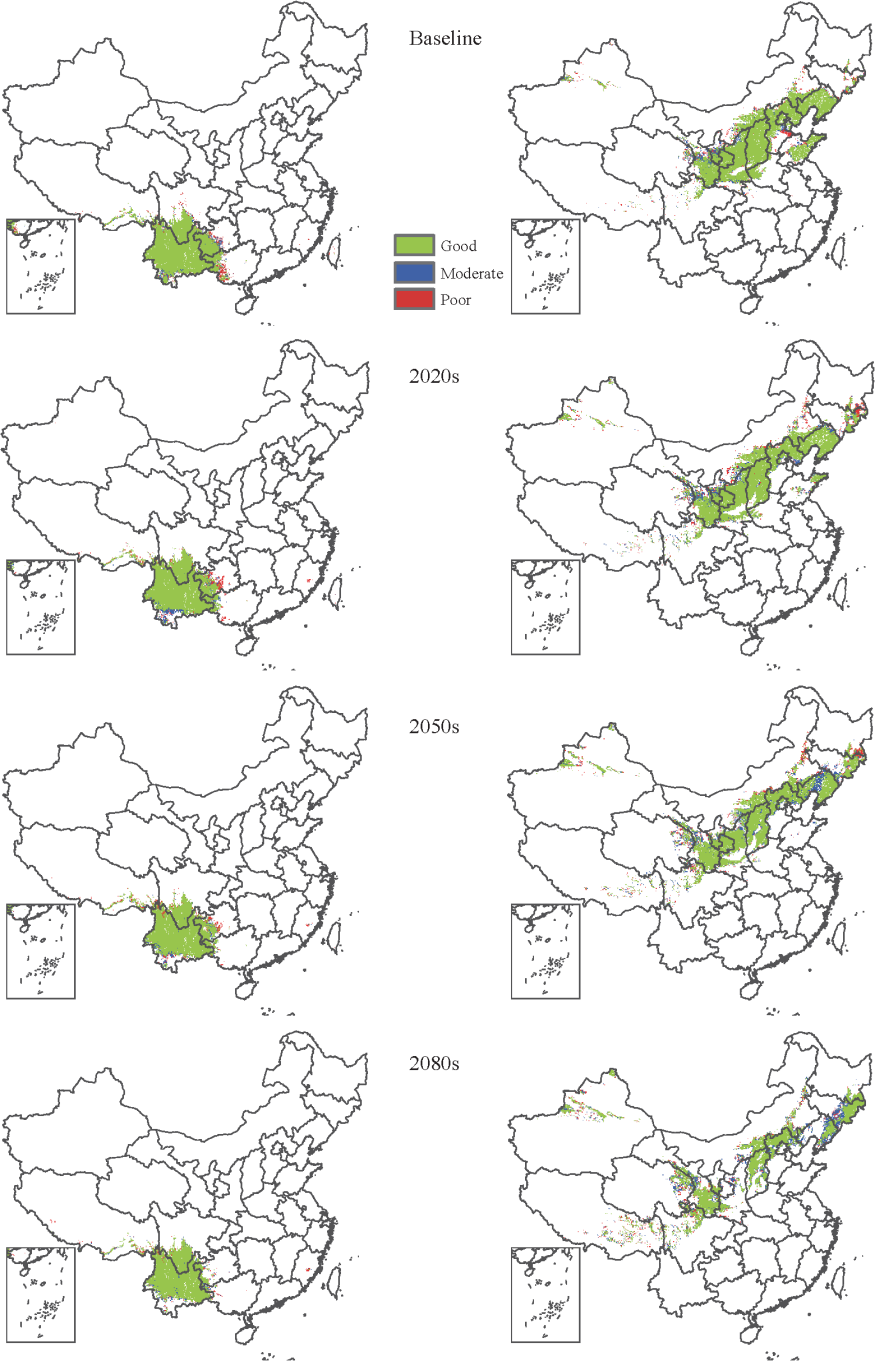
Overlap maps of current and future potential presence-absence distributions predicted using three different consensual approaches for *Pinus yunnanensis* (left column) and *Pinus tabulaeformis* (right column). Good (green) indicates species predicted to be present by all three consensus approaches. Moderate (blue) indicates species predicted to be present by any two of the three consensus approaches. Poor (read) indicates species predicted to be present by any one of the three consensus approaches.

The overlay maps of the three final consensual binary maps were produced for each species and each period (Fig. 3, S2 Fig.). Overlay analysis showed that, for most (but not all) species, the area of species occurrence collectively predicted by the three consensus approaches was located mainly in the core of the species range, while the incongruent area was located mainly at the edges of the species range or discrete locations (Fig. 3, S2 Fig.). For a majority of the 32 species, the ratio of incongruent to congruent area increased over time (S4 Table). For example, the ratio of incongruent to congruent area decreased with increasing time horizon for *Pinus yunnanensis*, but it increased with time for *Pinus tabulaeformis* (Fig. 3).

**Fig 4:**
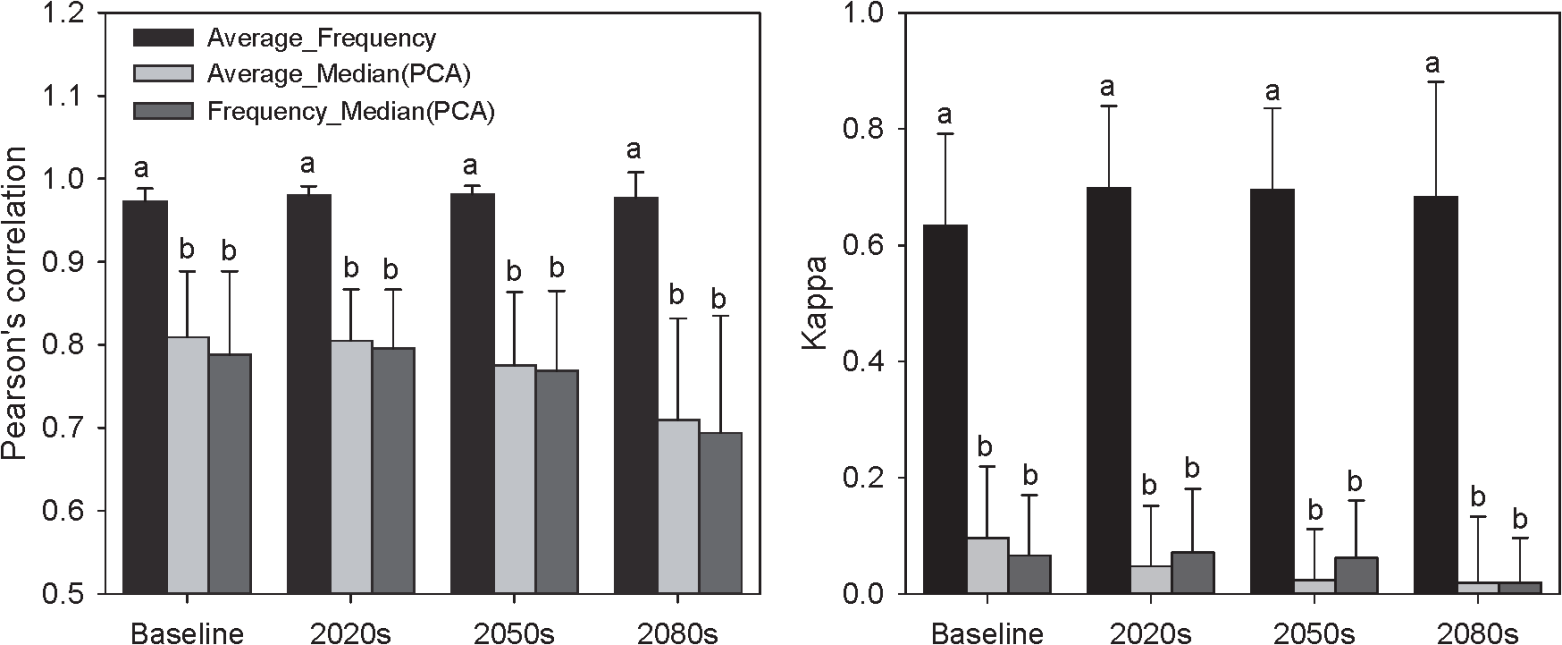
Pairwise correlation among predictions produced by three different consensual approaches (average, frequency, and median (PCA)). Data are presented as mean ± SE. Means in the same time slice followed by the same letter are not significantly different at *P* ≤ 0.05 according to LSD.

Mean correlation among consensual prediction maps varied according to the consensus approaches used to produce the maps, and there was substantial variability in the correlations among species (Fig. 4). For both consensual binary and probabilistic maps, analysis of variance showed that correlations were different (*P* < 0.001) among pairs for each time period.

When averaging the Pearson’s correlation coefficients across all trees species, the correlation between probabilistic maps between average and frequency was higher than that between median (PCA) and average or frequency (Fig. 4). Pairwise correlation analysis for the three consensual binary maps indicated that the correlation between average and frequency was also higher than that between median (PCA) and average or frequency (Fig. 4). The correlation between median (PCA) and average was not significantly different from that between median (PCA) and frequency in terms of either Kappa or Pearson’s correlation.

### Species traits and map correlation

When all of the explanatory variables (latitudinal, thermal, and elevation ranges, prevalence, specialization, marginality, AUC, Kappa, and TSS values) were included in multiple stepwise regression models, only Kappa, marginality, and specialization remained significant predictors, of map correlation (Table 4). Under both current and future climates, Pearson’s correlation increased with increasing Kappa and decreasing marginality. In future climates, specialization exerted a negative effect on the Pearson’s correlation. In the current climate, the map correlation represented by Kappa increased as the accuracy of the model increased (Kappa).

**Table 4:** Species traits, model accuracy, and map correlation.

Time	Map correlation	Variable	Coefficient	*P*-value
Baseline	Pearson’s correlation	(Constant)	0.542	< 0.001
		Kappa	0.418	< 0.001
		Marginality	−0.004	0.002
			R^2^ = 0.648	
	Kappa	(Constant)	0.170	< 0.001
		Kappa	0.115	0.033
			R^2^ = 0.143	
Future time	Pearson’s correlation	(Constant)	0.661	< 0.001
		Kappa	0.251	0.002
		Specialization	−6.273E-05	0.010
		Marginality	−0.003	0.033
			R^2^ = 0.427	

Coefficient and *P*-values of F-statstic for variables retained in the multiple stepwise regression models of map correlation for baseline and future time periods.

## Discussion

### Model-building datasets and model performance

Ideally, SDM models would be verified on an entirely independent dataset. Model evaluations should be performed in at least two different time periods undergoing climate change [24]. Alternatively, a model can be developed in one area and then evaluated using species distribution data outside the range of environments on which the original model is based (herein termed model transferability) [1]. However, these cases are rarely tested in predicting climate-induced range shifts. The split-sample method is commonly used to evaluate model performance when completely independent data are not available. In this work, model-training data served as one source of uncertainty in SDM. For each species and SDM model, predictive accuracy varied over different model-training and testing data sets. To build reliable SDM models, further research is needed on how to sample pseudo absences and select data-splitting schemes. A few researchers have proposed guidelines on how to generate pseudo absences suitable for use with different modeling techniques [13,33].

For each species, predictive accuracy varied from one model to another when using the same mode-training and testing data sets. Some models (e.g. MDA with AUC < 0.5) even failed to be calibrated, and others, such as RF, GBM, GAM, and GLM frequently produced models with better predictive accuracy (Fig. 2). This is because niche models have different model-building algorithms. There is considerable variation in extrapolating assumptions about the relationships between species and their environments [1,2,16]. Multiple-model comparison analysis with respect to predictive success indicated that more complex models tended to be more accurate [16]. As reported in previous studies, our results showed similar variations in predictive accuracy among individual species and modeling techniques. A few studies concluded that MC contributed the more variation to uncertainty in SDM than other sources of uncertainty did, and this could hide the effects of different climate change scenarios [6,11,12,21,22]. For this reason, more attention should be paid to niche models in SDM. The current primary objective was not to address the differences between single niche models but rather to focus on whether species traits influence spatial correspondence among consensual prediction maps.

### Niche properties and model performance

Species traits might affect the model performance due to a large range of variations in climatic and ecological requirements for these species, which makes it difficult to find a consistent relationship between species distribution and environmental conditions [20,21]. There are many available studies of the relationship between species traits and niche model predictive accuracy. Some studies have concluded that species with restricted distribution ranges tend to have more accurate model predictions than species with wide ranges (e.g. [16,18]). Grenouillet et al. (2011)[15] reported that species prevalence and latitudinal range had no significant effect on model performance, and more accurate predictions were obtained for species with low thermal and elevation ranges. Segurado and Araújo (2004)[26] noted that model performance was more pronounced for species with high environmental specialization and marginality than for generalist species. Species with small latitudinal and elevation ranges yielded models with higher predictive accuracy. This confirmed the hypothesis that generalist species yield models with lower accuracy than specialist ones. The predictive accuracy of consensus approaches was not evaluated here because numerous studies have already demonstrated that consensus approaches can substantially improve the predictive performance of single niche models (e.g. [7,11,12,24]). Grenouillet et al. (2011)[15] found that the predictive performance of ensemble forecasting was positively related to species prevalence and negatively related to thermal and elevation ranges.

### Niche properties and consensus forecasting

Although studies using ensemble forecasting to predict habitat suitability have identified areas of spatial uncertainty by comparing maps of projections [11,12,15,21], no maps of uncertainty emanating from different consensus approaches have been provided. The current study showed that the agreement between consensus approaches was spatially structured for all 32 species, with the congruent area mainly located in the core area within a species range and incongruent areas occurring primarily at the edge of species range or at discrete locations. Studies dealing with the predictive accuracy of consensus approaches have not directly evaluated the spatial similarity of consensual prediction maps [7,24], so comparisons to other taxonomic groups remain difficult. Nevertheless, a few studies have compared the spatial correlation among distribution maps derived from different single-niche models. For example, Grenouillet et al. (2011)[15] demonstrated that the most notable disagreement between predictions occurred at the edge of the recorded distributions of species, and species prevalence was positively related to the consensus among niche model predictions. Syphard and Franklin (2009)[40] showed that map correspondence was most pronounced when single-model prediction accuracy was high and prevalence was intermediate. In the current case, correlation among consensual prediction maps was positively related to the predictive performance of niche models and negatively related to species specialization and marginality. These findings emphasize that significant improvement in the reliability of consensus approaches can be achieved using niche models with high predictive accuracy. These improvements were more pronounced for species with low marginality and specialization than specialist ones.

Species traits can substantially influence the vulnerability of range changes to environmental changes [5,41,42]. The most vulnerable plant species are those with a restricted distribution [20,41]. Both niche models and climate scenarios (i.e.GCMs, SRESs) have dramatic discrepancies in forecasting species range shifts and extinction rates under altered climate conditions [2,21,22,24,43]. Then the differences in the prediction maps may become more apparent in ensemble forecasting for specialist species than for generalist species under climate change conditions. Considering the differences in the way of developing consensus approaches to derive the final consensual prediction maps (see below), it is here speculated that map correlation among consensus approaches should be high for generalist species. This speculation is consistent with current observations that map correlations were high for species with low specialization and marginality. To generalize strong results, additional investigations are needed to better evaluate both intra- and inter-taxonomic group variabilities in spatial correspondence among consensual predictions. Given the limitations of niche model and the general conclusion that specialist species more often yield models with high predictive accuracy, it is here argued that developing new single, better niche model with better model transferability is needed for better prediction of species distribution using consensus approaches.

### Consensus forecasting and model selection

Projections vary among models. One response to this is to build a set of models across more than one set of IC, MC, MP, and BC combinations for analysis of the range of predictions and achievement of consensus among different predictions. The reason to use consensus approach is based on the central limit theorem in statistics [24]. Consensus forecasting will not necessarily provide the most accurate future projection but may at least provide the most conservative future projection [6,12]. It therefore appears to have the greatest potential for predicting species range shifts in the context of climate change by identifying the most plausible direction and magnitude of range shifts of species. RF and GBM have been shown to be more robust than other commonly used approaches [2,15]. This was also found to be the case here, probably because they both inherently incorporated the concept of ensemble forecasting [10, 15,18].

In the current study, none of the three consensus approaches performed equally well in projecting species distribution range onto a future scenario. A non-significant difference in species’ relative range changes among the three consensus approaches did not reflect the spatial correspondence among the distribution maps derived from the three consensus approaches. Both in terms of probabilistic and binary maps, there was a substantial difference between three consensus approaches. Although a few studies have compared the predictive performances of different consensus approaches (e.g. [7,24]), none has, to our knowledge, discussed the reasons for the observed differences among consensual projections. It is here suspected that spatial divergence among prediction maps may be related to the method by which ensembles of forecasting were combined. The way consensus approaches behave under combined forecasting can differ. The median method is less sensitive to outliers than the average method. Frequency is inseparably linked to the threshold used to transform probability of species occurrence into binary map. The choice of threshold can also influence species range change predictions [14,24,44]. The three consensus approaches implemented in this study were based on the outputs of all single-predictions, while other combinative algorithms have been proposed to preselect the single-models based on certain predefined criteria [7,22]. There is still debate on the best methodology for combining model projections. To advance the improvement of ensemble forecasting framework in SDM, consensus approaches must be comprehensively evaluated and it must be determined whether simple consensus approaches perform as well as more complicated approaches. Maps produced by SDM are a fundamental component of conservation planning and resource management. From a conservation perspective, ensemble modeling and consensus approaches are expected to see increasingly common use for decision-making in resource management and designation of land in conservation planning. Incongruent areas should receive the most focus.

Ensemble forecasting assists the recent efforts to capitalize on the growing availability of species occurrence records, modeling techniques, and future climate scenarios. Although a wide spectrum of modeling approaches and GCM predictions based on alternative emissions scenarios exist, it does not necessarily mean that they should be incorporated in ensemble forecasting. Araújo et al. (2005)[24] argued that improved predictive accuracy still depends on traditional practices of building better models with improved data. Grenouillet et al. (2011)[15] and Crimmins et al. (2013) [45] further indicated that consensus forecasting method will not always outperform single models. Current results demonstrated that spatial correspondence among consensual prediction maps could be improved by using niche models with high predictive accuracy. The individual models must be as accurate and diverse as possible if the consensus forecast is to be accurate or effective [46]. The term “individual model” generally refers to the sub-models with the same mathematical and statistical properties (e.g. the sub-model of RF and GBM is classification tree, and they use internal validation to derive their model set). Predicting the effects of climate change on species distribution using ensemble forecasting frameworks is complicated, it usually requires both kinds of models, including niche models with more than one model-building algorithms, and climate models with different physical process and SRES emission scenarios. A few researchers have demonstrated that future climate scenarios show considerable variation in SDM, almost as much as niche models [12,21,22]. Climate models are complex tools: variability occurs among alternate simulations. No single model has been recognized as best. Identifying and selecting the most appropriate GCMs and SRESs is one way to reduce uncertainty in climate scenarios [47]. As such, it is likely that minimizing known uncertainties in SDM based on existing knowledge may improve reliability in the future projections by means of consensus forecasting. However, consensual forecasting in SDM is still in its infancy and more efforts are required to assess their strengths and weaknesses and those of ensemble schemes.

## Conclusions

The present study provides conceptual insights regarding the uncertainty of modeling the response of species to climate change. Results support previous findings that specialist species have more accurate results under modeling than generalist species and that model robustness is related to model complexity. These findings, along with those showing that different modeling techniques show various degrees of susceptibility to model-training data, could have important implications for preselecting models in SDM. Spatial uncertainty in ensemble forecasting of species distributions was here found to be related to the accuracy of single models and the positions of species in ecological space. The finding that generalist species yield distribution maps with lower spatial uncertainty than specialist species in projecting their distributions under current and future climate conditions could have important implications for consensus forecasting of species distributions. The positive relationship between spatial correspondence among consensual predictions and model performance suggests that additional efforts should be made to select or develop a new niche model with high spatial-temporal transferability. We conclude that species niche properties and model performance should be taken into account more critically in ensemble forecasting of species distributions, and particularly in the assessments of climate change impacts.

## Supporting Information

S1 Table Ecological requirements and biological characteristics of the 32 tree species used in this study. (DOC)

S2 Table Variation coefficients of niche model accuracies. (DOC)

S3 Table Predicted changes in species distribution range. (DOC)

S4 Table Consistency ratio of species distribution maps derived from three consensus approaches. (DOC)

S1 Fig Box-whisker plot showing predictive accuracy (AUC, Kappa, and TSS) for each species when data were pooled for all models. (DOC)

S2 Fig Overlap maps for current and future distribution as predicted using consensus approaches. (DOC)

S1 Appendix Description of the software package ClimateChina. (DOC)
