# Supplementary material for: Consensus Forecasting of Species Distributions: The Effects of Niche Model Performance and Niche Properties
Source: PLoS One. 2015 Mar 18;10(3):e0120056. doi: 10.1371/journal.pone.0120056 (PMC4364626; doi:10.1371/journal.pone.0120056)
Supplement: S1 Table — (DOC) [file pone.0120056.s001.doc]

# Table S1. Ecological requirements and biological characteristics of the 32 tree species used in this study.

Thirty-two tree species were used in the modeling analysis. The species’ ecological requirements and biological characteristics are abbreviated as follow: MAT=mean annual temperature; MAP=mean annual precipitation; E=evergreen; D=deciduous; H=humid [area](http://www.iciba.com/area/); SH=semi-humid [area](http://www.iciba.com/area/); A=arid area; SA=semi-arid [area](http://www.iciba.com/area/); B=broad-leaved; N=needle-leaved; S=scale-leaved.

| **Scientific name** | **Latitude/longitude range** | **Elevation range (m)** | **MAT range (°C)** | **MAP range (mm)** | **Habitat type** | **Leaf longevity** | **Leaf type** | **China endemic species** |
| --- | --- | --- | --- | --- | --- | --- | --- | --- |
| *Castanopsis fargesii* | N: 22.1–32.1°  E: 102.4–121.8° | 50–2900 | 8.2–23.5 | 690–4700 | H | E | B | No |
| *Castanopsis hystrix* | N: 21.1–30.1°  E: 87.3–121.5° | 50–4350 | 1.6–24 | 400–8400 | H | E | B | No |
| *Castanopsis sclerophylla* | N: 24.8–33.8° E: 104.4–121.9° | 2–2601 | 5.8–19.9 | 589–2629 | H | E | B | Yes |
| *Cunninghamia lanceolata* | N: 22–34°  E: 102–122° | 800–1800 | 12–23 | 800–2000 | H | E | N | Yes |
| *Davidia involucrata* | N: 24.9–34.2°  E: 98.7–111.9° | 200–4069 | 2.6–17.6 | 785–2156 | H | E | B | Yes |
| *Fraxinus mandschurica* | N: 41–51.9°  E: 120.6–132° | 96–1470 | -5.2–6.2 | 459–1060 | SA-SH | D | B | No |
| *Larix gmelinii* | N: 44–53°  E: 119–133° | 300–1200 | -6–0 | 350–600 | H-SH | D | N | No |
| *Larix olgensis* | N: 40–48°  E: 123–131° | 500–1800 | 2–6.4 | 540–1200 | H-SH | D | N | No |
| *Larix principis-rupprechtii* | N: 36–42°  E: 111–124° | 1200–2800 | 2–12 | 350–800 | SH-SA | D | N | Yes |
| *Phyllostachys edulis* | N: 23.6–31.9° E: 104.9–121.8° | 5–1265 | 10.8–21.1 | 1050–2753 | H-SH | E | B | Yes |
| *Picea asperata* | N: 28–35°  E: 99–106° | 2400–3600 | 2–10 | 500–1100 | SH-SA | E | N | Yes |
| *Picea crassifolia* | N: 32.4–39.6°  E: 97.9–106.3° | 1352–4396 | -4.1–12.3 | 190–1050 | SA-SH | E | N | Yes |
| *Picea likiangensis* | N: 26.4–30°  E: 99–102.1° | 1588–4946 | -4.4–14.5 | 410–1900 | H | E | N | Yes |
| *Picea schrenkiana* | N: 37–46°  E: 74.9–94.8° | 999–4098 | -6.3–8.1 | 80–1001 | SA | E | N | No |
| *Pinus armandii* | N: 25–36°  E: 101–112° | 1000–3200 | 4–18 | 500–1500 | H-SH-SA | E | N | Yes |
| *Pinus koraiensis* | N: 41–51°  E: 123–134° | 300–1200 | -1.6–8.5 | 500–1200 | H-SH | E | N | No |
| *Pinus massoniana* | N: 21–34°  E: 103–122° | 600–1500 | 11.5–22.6 | 754–2179 | H | E | N | Yes |
| *Pinus sylvestris* var*. mongolica* | N: 41–53°  E: 119–130° | 400–900 | -6–8 | 300–700 | H-SH | E | N | No |
| *Pinus tabulaeformis* | N: 31–43°  E: 100–126° | 500–1500 | 2–14 | 200–1000 | SH-SA | E | N | Yes |
| *Pinus yunnanensis* | N: 22–29°  E: 98–107° | 1000–3200 | 11–20.9 | 800–1500 | H | E | N | Yes |
| *Platycladus orientalis* | N: 29–40°  E: 104–119° | 1000–3600 | 2–16 | 200–1200 | SH | E | S | Yes |
| *Populus davidiana* | N: 25–53.1° E: 84.4–130.4° | 100–5251 | -4.4–18 | 236–1738 | SA-SH | D | B | No |
| *Populus euphratica* | N: 36.3–44.5° E: 77.4–111.3° | 390–2918 | 2.1–11.7 | 33–384 | A-SA | D | B | No |
| *Quercus acutissima* | N: 23.5–40.7° E: 104.1–125.4° | 9–2737 | 4.9–22.2 | 546–2089 | H-SH | D | B | No |
| *Quercus fabri* | N: 23.6–35.9° E: 102.2–122° | 0–3349 | 4.9–20.1 | 584–2489 | H | D | B | No |
| *Quercus liaotungensis* | N: 32.6–43.2° E: 102.9–125.6° | 27–4187 | -0.9–13.4 | 448–1285 | H-SH | D | B | No |
| *Quercus mongolica* | N: 35.6–52.8° E: 108.3–134.5° | 45–1818 | -4.7–10.7 | 361–1216 | H-SH | D | B | No |
| *Quercus variabilis* | N: 23.5–40.01° E: 99.1–123° | 0–4530 | -1.5–22.7 | 424–2393 | H-SH | D | B | No |
| *Taiwania cryptomerioides* | N: 22.4–30.4° E: 98–121.5° | 100–3720 | 5.6–23.1 | 981–5927 | H | E | N | No |
| *Tilia amurensis* | N: 40.8–51.6° E: 124.3–135.0° | 40–1500 | -2.3–6 | 470–1137 | H-SH | D | B | No |
| *Tilia mandshurica* | N: 40–48.4° E: 122.7–134.5° | 40–1488 | -1.4–7.3 | 500–1137 | SA-SH | D | B | No |
| *Tsuga dumosa* | N: 23.6–29.5° E: 86.0–101.7° | 470–4956 | -2.7–21.1 | 340–7909 | H-SH | E | N | No |
