# Supplementary material for: Consensus Forecasting of Species Distributions: The Effects of Niche Model Performance and Niche Properties
Source: PLoS One. 2015 Mar 18;10(3):e0120056. doi: 10.1371/journal.pone.0120056 (PMC4364626; doi:10.1371/journal.pone.0120056)
Supplement: S2 Table — (DOC) [file pone.0120056.s002.doc]

# Table S2. Variation coefficients of niche model accuracy.

| Niche models | Coefficient of variation | | | | |
| --- | --- | --- | --- | --- | --- |
|  | AUC |  | KAPPA |  | TSS |
| ANN | 0.019(0.017)a |  | 0.134(0.123)a |  | 0.046(0.038)ab |
| CTA | 0.012(0.010)a |  | 0.050(0.063)b |  | 0.022(0.019)a |
| GAM | 0.002(0.003)a |  | 0.034(0.044)b |  | 0.012(0.008)a |
| GBM | 0.002(0.002)a |  | 0.032(0.037)b |  | 0.012(0.008)a |
| GLM | 0.003(0.003)a |  | 0.038(0.050)b |  | 0.012(0.009)a |
| MARS | 0.051(0.095)b |  | 0.122(0.165)a |  | 0.083(0.140)b |
| MDA | 0.169(0.113)b |  | 0.491(0.336)c |  | 0.363(0.259)c |
| RF | 0.002(0.003)a |  | 0.024(0.024)b |  | 0.012(0.009)a |

Note: Values are means of 32 samples (tree species) and standard errors are presented in parentheses. Each sample is defined as the variation coefficient of the predictive accuracy of a single niche model as indicated by nine split-sample bouts (i.e. original data were randomly divided into two sets: a calibration set and a validation set). Means in a column followed by the same letter are not significantly different at *P* ≤0.05 according to LSD. ANN, artificial neural network; CTA, classification tree analysis; GAM, generalized additive model; GBM, generalized boosting method; GLM, generalized linear model; MARS, multivariate adaptive regression spline, MDA, mixture discriminant analysis; RF, random forest.
