# Supplementary material for: Consensus Forecasting of Species Distributions: The Effects of Niche Model Performance and Niche Properties
Source: PLoS One. 2015 Mar 18;10(3):e0120056. doi: 10.1371/journal.pone.0120056 (PMC4364626; doi:10.1371/journal.pone.0120056)
Supplement: S3 Table — (DOC) [file pone.0120056.s003.doc]

# Table S3 Species range changes under future climate.

Changes in the distribution range of tree species (change in area and shift in distance and direction of mean centers of suitable habitat) as indicated by consensus approaches (average, frequency, and median (PCA)) for the normal periods 2010–2039 (2020s), 2040–2069 (2050s), and 2070–2099 (2080s) relative to current baseline (1961–1990).

| ***Scientific name*** | Consensus | Total range change (%) | | |  | Eastward shift (km) | | |  | Northward shift (km) | | |
| --- | --- | --- | --- | --- | --- | --- | --- | --- | --- | --- | --- | --- |
|  |  | 2020s | 2050s | 2080s |  | 2020s | 2050s | 2080s |  | 2020s | 2050s | 2080s |
| *Castanopsis fargesii* | Average | 5.5 | 13.0 | 19.9 |  | 0.6 | 0.9 | 1.6 |  | 44.7 | 67.0 | 111.7 |
|  | Frequency | 4.8 | 12.5 | 20.8 |  | 1.0 | -7.8 | -16.4 |  | 78.0 | 145.1 | 223.5 |
|  | Median(PCA) | -1.3 | -0.8 | -1.4 |  | -10.0 | -9.9 | 0.2 |  | 0.1 | 11.2 | 22.2 |
| *Castanopsis hystrix* | Average | 5.2 | 6.9 | 6.6 |  | 11.5 | 12.5 | 23.2 |  | 55.6 | 89.2 | 122.4 |
|  | Frequency | 3.6 | 5.1 | 3.7 |  | 3.7 | 14.0 | 14.8 |  | 111.8 | 133.9 | 156.3 |
|  | Median(PCA) | -1.9 | -3.6 | -9.4 |  | -9.7 | -19.7 | -59.9 |  | 11.4 | 11.8 | 13.3 |
| *Castanopsis sclerophylla* | Average | -3.3 | -8.8 | -15.5 |  | -19.1 | -47.5 | -85.2 |  | 22.3 | 44.6 | 67.0 |
|  | Frequency | -4.6 | -10.6 | -17.6 |  | 28.8 | 9.7 | -94.5 |  | -33.3 | -22.4 | 44.4 |
|  | Median(PCA) | -2.7 | -4.5 | -10.3 |  | -0.7 | -0.3 | -9.6 |  | 22.4 | 11.2 | -0.3 |
| *Cunninghamia lanceolata* | Average | -1.1 | -1.2 | -2.3 |  | -19.6 | -39.2 | -68.4 |  | 22.2 | 55.6 | 104.3 |
|  | Frequency | -1.4 | -0.4 | -0.4 |  | -19.9 | -60.3 | -148.9 |  | 10.7 | 77.0 | 182.6 |
|  | Median(PCA) | -2.4 | -1.4 | -1.0 |  | -9.8 | -9.8 | -19.9 |  | -0.2 | -0.2 | 13.9 |
| *Davidia involucrata* | Average | -25.1 | -40.8 | -57.0 |  | -27.5 | -45.6 | -53.7 |  | 23.4 | 46.5 | 80.6 |
|  | Frequency | -25.7 | -41.8 | -58.4 |  | -39.3 | -47.9 | -29.9 |  | -43.6 | -20.7 | -43.9 |
|  | Median(PCA) | -26.1 | -42.0 | -58.8 |  | -75.3 | -130.8 | -194.7 |  | 47.4 | 94.6 | 153.8 |
| *Fraxinus mandschurica* | Average | -19.9 | -42.3 | -71.4 |  | -151.0 | -264.7 | -447.7 |  | -111.6 | -196.2 | -337.4 |
|  | Frequency | -19.8 | -41.6 | -71.6 |  | -258.8 | -611.7 | -778.7 |  | -230.8 | -558.9 | -654.7 |
|  | Median(PCA) | -16.0 | -35.5 | -61.3 |  | -40.2 | -77.2 | -132.9 |  | 5.3 | -11.4 | -7.8 |
| *Larix gmelinii* | Average | -26.3 | -39.1 | -68.7 |  | -179.2 | -347.4 | -556.4 |  | -146.5 | -311.3 | -552.1 |
|  | Frequency | -27.5 | -40.1 | -68.4 |  | -197.3 | -629.9 | -898.1 |  | -143.9 | -665.7 | -978.5 |
|  | Median(PCA) | -28.5 | -42.4 | -66.1 |  | -107.7 | -148.5 | -197.2 |  | 73.1 | 100.7 | 135.2 |
| *Larix olgensis* | Average | 9.5 | 2.2 | -17.2 |  | -24.7 | -74.4 | -166.1 |  | -12.8 | -38.1 | -88.1 |
|  | Frequency | 7.7 | -0.7 | -15.5 |  | 190.4 | 251.6 | -185.2 |  | 157.2 | 255.9 | -92.4 |
|  | Median(PCA) | 15.1 | 4.0 | -6.4 |  | 54.2 | 46.3 | 28.8 |  | 66.0 | 64.7 | 71.9 |
| *Larix principis-rupprechtii* | Average | -42.8 | -64.0 | -86.0 |  | -71.0 | -143.0 | -234.7 |  | -44.2 | -99.1 | -178.3 |
|  | Frequency | -45.3 | -64.5 | -84.0 |  | -101.8 | -307.8 | -791.1 |  | 75.7 | 95.5 | -474.2 |
|  | Median(PCA) | -38.9 | -57.1 | -80.7 |  | -50.6 | -117.4 | -269.3 |  | 10.1 | -13.5 | -110.2 |
| *Phyllostachys edulis* | Average | -4.2 | -17.3 | -51.0 |  | -67.6 | -124.8 | -209.5 |  | 66.4 | 110.9 | 189.6 |
|  | Frequency | -8.4 | -23.7 | -55.9 |  | -117.1 | -193.6 | -324.8 |  | 109.9 | 176.5 | 300.5 |
|  | Median(PCA) | -4.2 | -17.3 | -51.0 |  | 19.5 | 0.0 | -39.1 |  | 0.7 | 0.0 | -1.4 |
| *Picea asperata* | Average | 4.7 | 17.5 | 34.9 |  | -46.2 | -91.8 | -174.5 |  | -8.7 | -5.9 | -9.3 |
|  | Frequency | -3.1 | 6.8 | 18.7 |  | -45.7 | -110.3 | -127.5 |  | 2.6 | -4.5 | 13.3 |
|  | Median(PCA) | 3.6 | 15.2 | 30.6 |  | -119.6 | -211.0 | -339.7 |  | 22.1 | 42.8 | 61.8 |
| *Picea crassifolia* | Average | 22.9 | 55.1 | 107.4 |  | -144.5 | -236.0 | -347.0 |  | -10.1 | -23.7 | -45.4 |
|  | Frequency | 17.7 | 45.5 | 96.1 |  | -42.6 | -196.3 | -389.2 |  | -63.6 | -68.3 | -87.4 |
|  | Median(PCA) | 25.8 | 55.0 | 82.4 |  | -189.0 | -283.5 | -398.3 |  | -10.7 | -43.1 | -82.9 |
| *Picea likiangensis* | Average | -10.1 | -13.1 | -20.5 |  | -17.8 | -35.5 | -72.4 |  | 12.4 | 24.8 | 27.3 |
|  | Frequency | -11.3 | -13.9 | -20.0 |  | -7.8 | -17.0 | -91.4 |  | 23.1 | 23.6 | 17.3 |
|  | Median(PCA) | -10.9 | -15.3 | -22.4 |  | -45.3 | -63.2 | -106.9 |  | 27.1 | 40.3 | 78.9 |
| *Picea schrenkiana* | Average | -0.6 | -5.3 | -13.9 |  | -73.6 | -105.1 | -119.2 |  | -26.0 | -67.9 | -111.7 |
|  | Frequency | -4.2 | -8.9 | -18.9 |  | -9.3 | -27.3 | -63.0 |  | -67.2 | -133.2 | -196.6 |
|  | Median(PCA) | 9.1 | 15.2 | 20.1 |  | -9.3 | -27.3 | -63.0 |  | -67.2 | -133.2 | -196.6 |
| *Pinus armandii* | Average | -19.2 | -31.3 | -38.9 |  | -45.6 | -95.7 | -199.9 |  | 24.3 | 60.3 | 88.4 |
|  | Frequency | -21.4 | -33.7 | -43.5 |  | -45.5 | -107.4 | -256.2 |  | 35.7 | 61.1 | 115.1 |
|  | Median(PCA) | -18.3 | -30.6 | -36.4 |  | -72.8 | -163.9 | -354.8 |  | 59.5 | 109.4 | 182.8 |
| *Pinus koraiensis* | Average | -29.4 | -49.1 | -81.4 |  | -136.2 | -282.3 | -489.9 |  | -77.6 | -175.8 | -358.9 |
|  | Frequency | -32.7 | -52.5 | -83.7 |  | -110.7 | -243.0 | -456.2 |  | -24.0 | -82.2 | -310.6 |
|  | Median(PCA) | -35.8 | -35.0 | -40.3 |  | -68.9 | -69.3 | -143.4 |  | 34.2 | 135.9 | 314.4 |
| *Pinus massoniana* | Average | 3.8 | 6.0 | 9.7 |  | -9.8 | -19.5 | -29.2 |  | 33.6 | 55.9 | 82.2 |
|  | Frequency | 4.1 | 6.1 | 9.5 |  | -10.1 | -59.1 | -88.1 |  | 33.4 | 111.3 | 160.8 |
|  | Median(PCA) | 3.7 | 4.7 | 5.3 |  | -0.2 | -10.2 | -20.1 |  | 11.2 | 22.1 | 31.9 |
| *Pinus sylvestris* var*. mongolica* | Average | -30.9 | -56.9 | -76.2 |  | -91.8 | -151.2 | -211.6 |  | -92.2 | -172.0 | -256.6 |
|  | Frequency | -15.7 | -35.7 | -56.1 |  | -708.6 | -891.8 | -1013.5 |  | -932.8 | -1162.4 | -1306.8 |
|  | Median(PCA) | -9.6 | -22.8 | -58.8 |  | -13.8 | 9.5 | 147.9 |  | -12.9 | -77.4 | -9.5 |
| *Pinus tabulaeformis* | Average | -4.0 | -6.5 | -24.0 |  | -52.5 | -104.9 | -192.8 |  | 11.4 | 11.7 | -13.5 |
|  | Frequency | -3.5 | -6.5 | -23.2 |  | -78.6 | -157.8 | -246.2 |  | -0.2 | -44.9 | -72.3 |
|  | Median(PCA) | -3.1 | -10.6 | -31.6 |  | -26.7 | -86.9 | -250.3 |  | 56.1 | 66.8 | 35.7 |
| *Pinus yunnanensis* | Average | -6.9 | -11.5 | -21.8 |  | 1.9 | 2.6 | 34.8 |  | 33.5 | 44.7 | 106.3 |
|  | Frequency | -8.1 | -13.0 | -23.0 |  | 2.3 | -7.6 | 70.3 |  | 33.4 | 34.0 | 176.9 |
|  | Median(PCA) | -11.3 | -15.1 | -25.7 |  | -6.6 | -26.5 | -55.0 |  | 45.1 | 46.6 | 66.2 |
| *Platycladus orientalis* | Average | -20.1 | -27.1 | -40.8 |  | -125.3 | -187.7 | -241.2 |  | 11.4 | 23.4 | 28.6 |
|  | Frequency | -21.6 | -29.0 | -42.6 |  | -258.7 | -365.9 | -437.1 |  | -2.7 | -13.0 | -2.1 |
|  | Median(PCA) | -23.4 | -31.4 | -44.0 |  | -80.8 | -142.9 | -257.5 |  | 55.4 | 77.7 | 110.2 |
| *Populus davidiana* | Average | -17.3 | -28.5 | -38.7 |  | -135.7 | -307.2 | -624.6 |  | -36.2 | -115.2 | -264.2 |
|  | Frequency | -17.7 | -29.9 | -40.3 |  | -149.1 | -359.0 | -802.2 |  | -50.3 | -120.7 | -335.4 |
|  | Median(PCA) | -17.4 | -28.5 | -36.2 |  | -167.5 | -362.1 | -745.5 |  | -38.5 | -107.3 | -277.4 |
| *Populus euphratica* | Average | 12.0 | 18.6 | 22.1 |  | 36.9 | 90.4 | 170.8 |  | 19.8 | 27.3 | 44.7 |
|  | Frequency | 11.8 | 17.8 | 19.1 |  | 78.4 | 139.4 | 202.6 |  | -17.3 | -32.8 | -14.0 |
|  | Median(PCA) | 17.1 | 24.4 | 26.8 |  | 14.8 | 35.6 | 62.3 |  | 31.6 | 50.5 | 56.8 |
| *Quercus acutissima* | Average | -29.0 | -39.7 | -53.3 |  | -75.1 | -84.1 | -102.3 |  | 34.2 | 68.0 | 113.2 |
|  | Frequency | -31.0 | -42.5 | -56.3 |  | -75.8 | -85.1 | -94.1 |  | -11.0 | 11.5 | 56.6 |
|  | Median(PCA) | -25.9 | -35.7 | -48.2 |  | -104.0 | -103.7 | -103.5 |  | 22.4 | 78.6 | 123.6 |
| *Quercus fabri* | Average | -5.8 | -18.9 | -54.6 |  | -38.2 | -66.5 | -113.3 |  | 56.1 | 89.8 | 146.3 |
|  | Frequency | -6.3 | -19.8 | -54.0 |  | -77.4 | -106.0 | -181.2 |  | 122.8 | 134.0 | 224.3 |
|  | Median(PCA) | -6.9 | -24.0 | -53.0 |  | -10.0 | -58.3 | -183.5 |  | 22.2 | 32.9 | 43.8 |
| *Quercus liaotungensis* | Average | -5.7 | -19.8 | -42.7 |  | -35.4 | -88.5 | -204.1 |  | 22.6 | 11.7 | -9.1 |
|  | Frequency | -5.9 | -22.4 | -46.8 |  | 26.0 | -44.3 | -256.6 |  | 56.7 | 56.4 | -9.1 |
|  | Median(PCA) | -0.6 | -4.9 | -23.6 |  | 24.4 | -78.9 | -391.7 |  | 57.2 | 43.5 | -57.9 |
| *Quercus mongolica* | Average | -8.0 | -15.9 | -32.7 |  | -39.0 | -101.3 | -194.3 |  | -25.6 | -75.2 | -182.5 |
|  | Frequency | -8.5 | -17.1 | -33.7 |  | -43.6 | -170.4 | -208.7 |  | -49.4 | -184.4 | -221.2 |
|  | Median(PCA) | -9.5 | -20.6 | -33.1 |  | 6.0 | 12.1 | 30.9 |  | 12.1 | 24.3 | -51.9 |
| *Quercus variabilis* | Average | -15.0 | -23.5 | -35.6 |  | -74.9 | -112.1 | -167.2 |  | 45.7 | 68.8 | 115.0 |
|  | Frequency | -16.4 | -26.5 | -39.1 |  | -84.1 | -112.0 | -167.0 |  | 46.0 | 57.7 | 103.9 |
|  | Median(PCA) | -13.8 | -18.8 | -31.2 |  | -95.4 | -133.3 | -218.4 |  | 11.3 | 34.1 | 57.8 |
| *Taiwania cryptomerioides* | Average | -20.9 | -33.0 | -48.1 |  | -36.2 | -62.3 | -78.5 |  | 79.5 | 158.9 | 238.1 |
|  | Frequency | -19.9 | -22.4 | -31.2 |  | -65.5 | -113.3 | -133.1 |  | 157.5 | 494.9 | 732.0 |
|  | Median(PCA) | -14.0 | -27.6 | -44.9 |  | -48.6 | -77.7 | -106.7 |  | 23.7 | 35.9 | 48.1 |
| *Tilia amurensis* | Average | -28.7 | -46.9 | -73.1 |  | -140.0 | -279.6 | -504.9 |  | -31.7 | -94.9 | -236.3 |
|  | Frequency | -28.0 | -47.9 | -70.4 |  | -125.7 | -364.2 | -570.0 |  | 49.3 | -59.1 | -145.8 |
|  | Median(PCA) | -21.8 | -47.1 | -76.1 |  | -93.5 | -197.8 | -344.0 |  | 130.7 | 203.5 | 215.2 |
| *Tilia mandshurica* | Average | -20.1 | -40.5 | -68.4 |  | -99.6 | -247.7 | -474.1 |  | -17.6 | -92.6 | -245.4 |
|  | Frequency | -21.9 | -42.5 | -69.2 |  | -122.2 | -260.7 | -552.8 |  | -22.0 | -54.5 | -270.8 |
|  | Median(PCA) | -19.9 | -50.5 | -80.0 |  | -65.2 | -143.1 | -293.8 |  | 56.4 | 54.5 | 20.0 |
| *Tsuga dumosa* | Average | -18.1 | -26.9 | -38.3 |  | 2.6 | 4.5 | 25.7 |  | 44.9 | 78.6 | 122.5 |
|  | Frequency | -18.9 | -27.1 | -39.4 |  | 46.7 | 101.8 | 143.8 |  | 120.4 | 240.7 | 317.1 |
|  | Median(PCA) | -13.5 | -22.7 | -31.8 |  | -26.1 | -44.6 | -79.0 |  | 36.2 | 49.2 | 97.6 |
