# Supplementary material for: Consensus Forecasting of Species Distributions: The Effects of Niche Model Performance and Niche Properties
Source: PLoS One. 2015 Mar 18;10(3):e0120056. doi: 10.1371/journal.pone.0120056 (PMC4364626; doi:10.1371/journal.pone.0120056)
Supplement: S4 Table — (DOC) [file pone.0120056.s004.doc]

# Table S4 Consistency ratio of species distribution maps derived from three consensus approaches.

| Tree species | Ratio of incongruent to congruent area | | | |
| --- | --- | --- | --- | --- |
|  | Baseline | 2020s | 2050s | 2080s |
| *Castanopsis fargesii* | 0.096 | 0.097 | 0.122 | 0.178 |
| *Castanopsis hystrix* | 0.113 | 0.143 | 0.160 | 0.222 |
| *Castanopsis sclerophylla* | 0.111 | 0.088 | 0.146 | 0.161 |
| *Cunninghamia lanceolata* | 0.074 | 0.054 | 0.047 | 0.060 |
| *Davidia involucrata* | 0.189 | 0.176 | 0.169 | 0.189 |
| *Fraxinus mandschurica* | 0.157 | 0.300 | 0.588 | 0.922 |
| *Larix gmelinii* | 0.059 | 0.062 | 0.132 | 0.150 |
| *Larix olgensis* | 0.118 | 0.132 | 0.169 | 0.270 |
| *Larix principis-rupprechtii* | 0.742 | 0.759 | 0.941 | 1.279 |
| *Phyllostachys edulis* | 0.128 | 0.137 | 0.201 | 0.317 |
| *Picea asperata* | 0.377 | 0.334 | 0.312 | 0.277 |
| *Picea crassifolia* | 0.222 | 0.466 | 0.471 | 0.377 |
| *Picea likiangensis* | 0.349 | 0.380 | 0.382 | 0.481 |
| *Picea schrenkiana*. | 0.325 | 0.534 | 0.695 | 0.965 |
| *Pinus armandii* | 0.316 | 0.360 | 0.443 | 0.567 |
| *Pinus koraiensis* | 0.149 | 0.291 | 0.367 | 2.539 |
| *Pinus massoniana* | 0.025 | 0.029 | 0.038 | 0.068 |
| *Pinus sylvestris* var*. mongolica* | 0.771 | 1.054 | 1.782 | 2.257 |
| *Pinus tabulaeformis* | 0.142 | 0.209 | 0.292 | 0.389 |
| *Pinus yunnanensis* | 0.120 | 0.138 | 0.117 | 0.103 |
| *Platycladus orientalis* | 0.136 | 0.138 | 0.147 | 0.188 |
| *Populus davidiana* | 0.134 | 0.167 | 0.187 | 0.235 |
| *Populus euphratica* | 0.148 | 0.092 | 0.088 | 0.148 |
| *Quercus acutissima* | 0.180 | 0.152 | 0.205 | 0.342 |
| *Quercus fabri* | 0.176 | 0.126 | 0.249 | 0.350 |
| *Quercus liaotungensis* | 0.271 | 0.247 | 0.325 | 0.493 |
| *Quercus mongolica* | 0.084 | 0.077 | 0.079 | 0.290 |
| *Quercus variabilis* | 0.273 | 0.261 | 0.242 | 0.273 |
| *Taiwania cryptomerioides* | 1.283 | 1.386 | 1.398 | 1.473 |
| *Tilia amurensis* | 0.090 | 0.171 | 0.179 | 0.446 |
| *Tilia mandshurica* | 0.169 | 0.200 | 0.187 | 0.683 |
| *Tsuga dumosa* | 0.205 | 0.244 | 0.216 | 0.288 |

Note: Incongruent area refers to places where species are predicted to be present by only one or two of the three consensus approaches. Congruent areas are places where species are predicted to be present by all three consensus approaches.
