# Supplementary material for: Consensus Forecasting of Species Distributions: The Effects of Niche Model Performance and Niche Properties
Source: PLoS One. 2015 Mar 18;10(3):e0120056. doi: 10.1371/journal.pone.0120056 (PMC4364626; doi:10.1371/journal.pone.0120056)

# Figure S1 Box-whisker plot of predictive accuracy (AUC, Kappa, and TSS) for each species when data from all models were pooled.


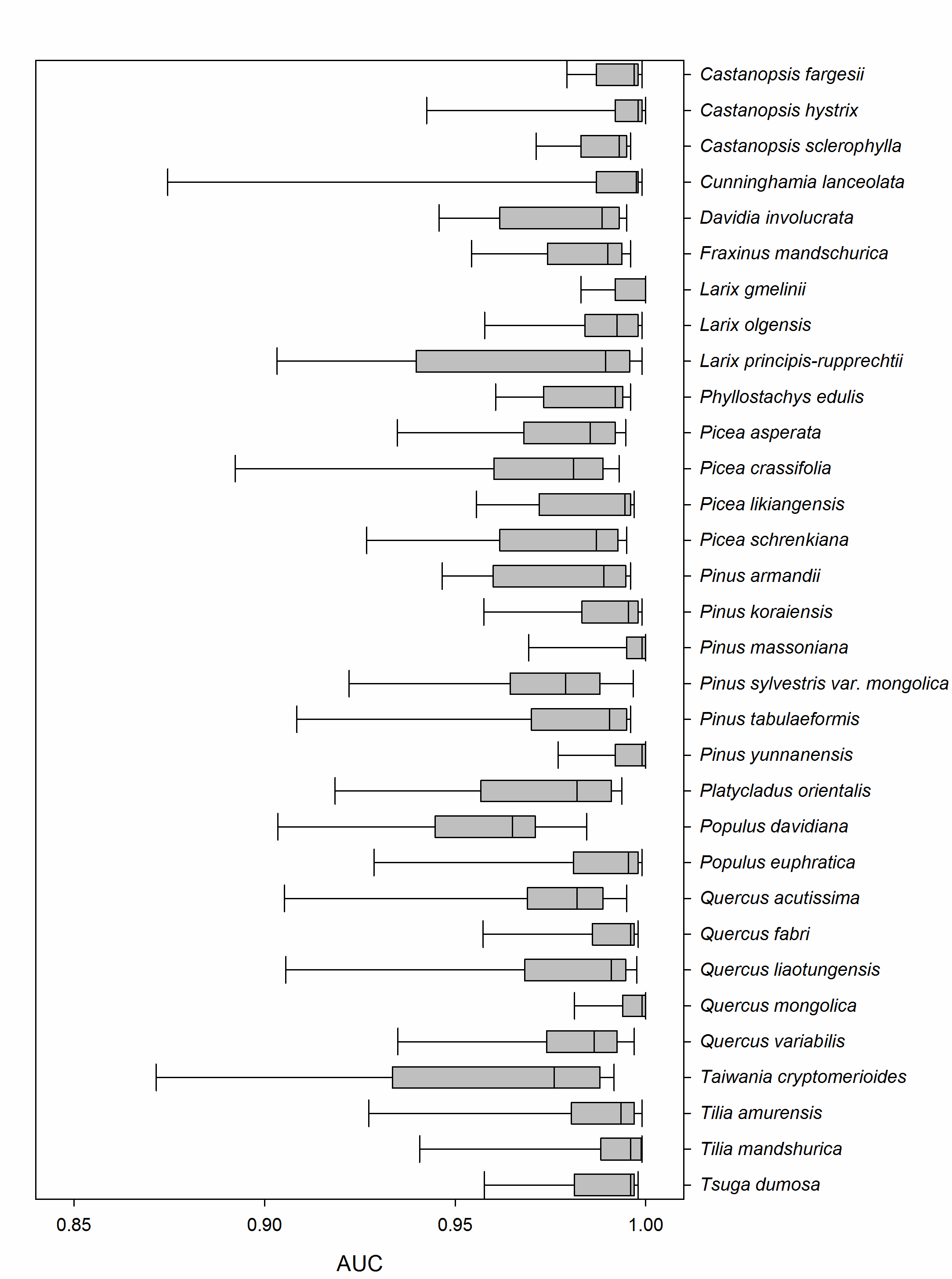


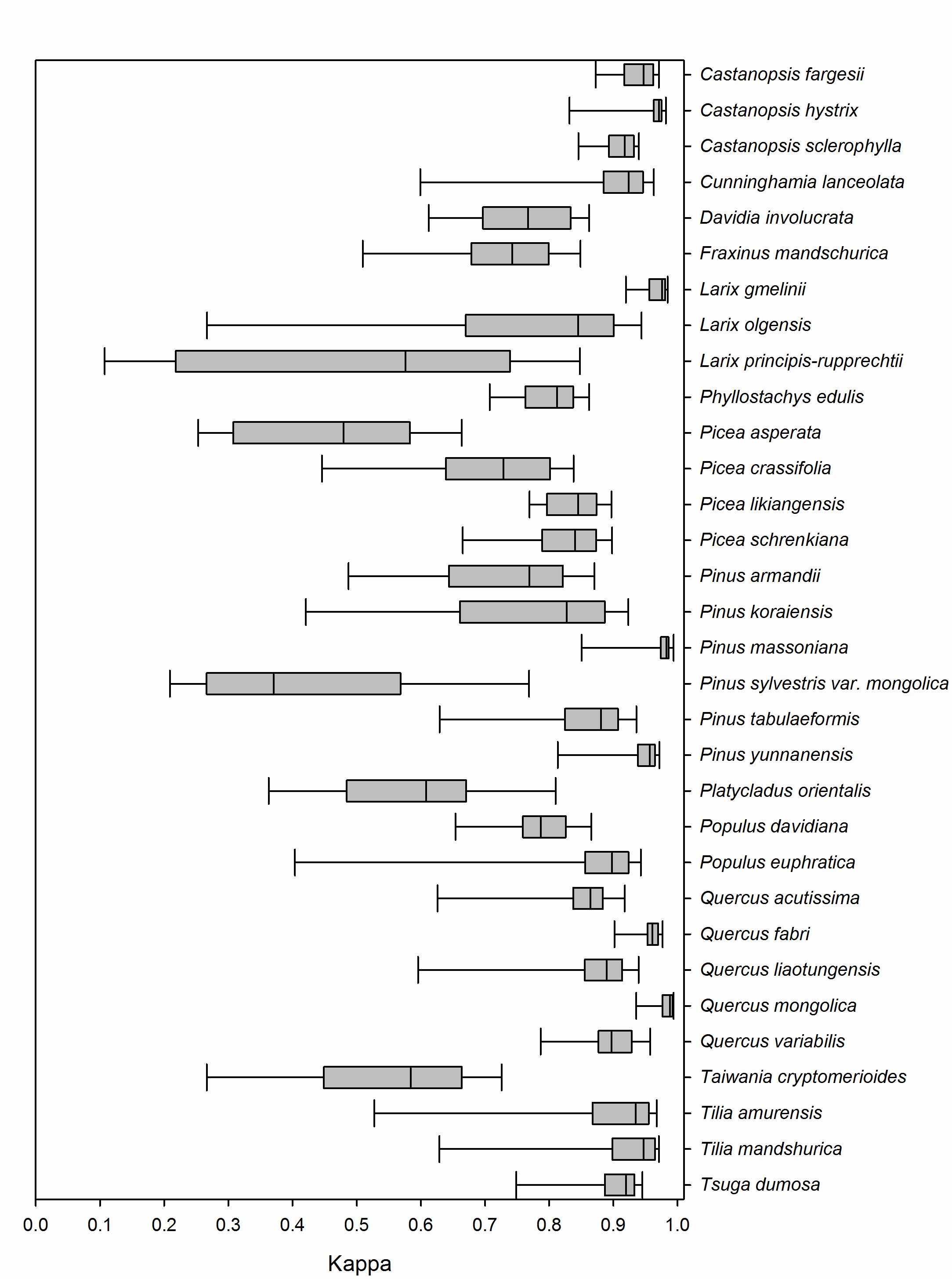


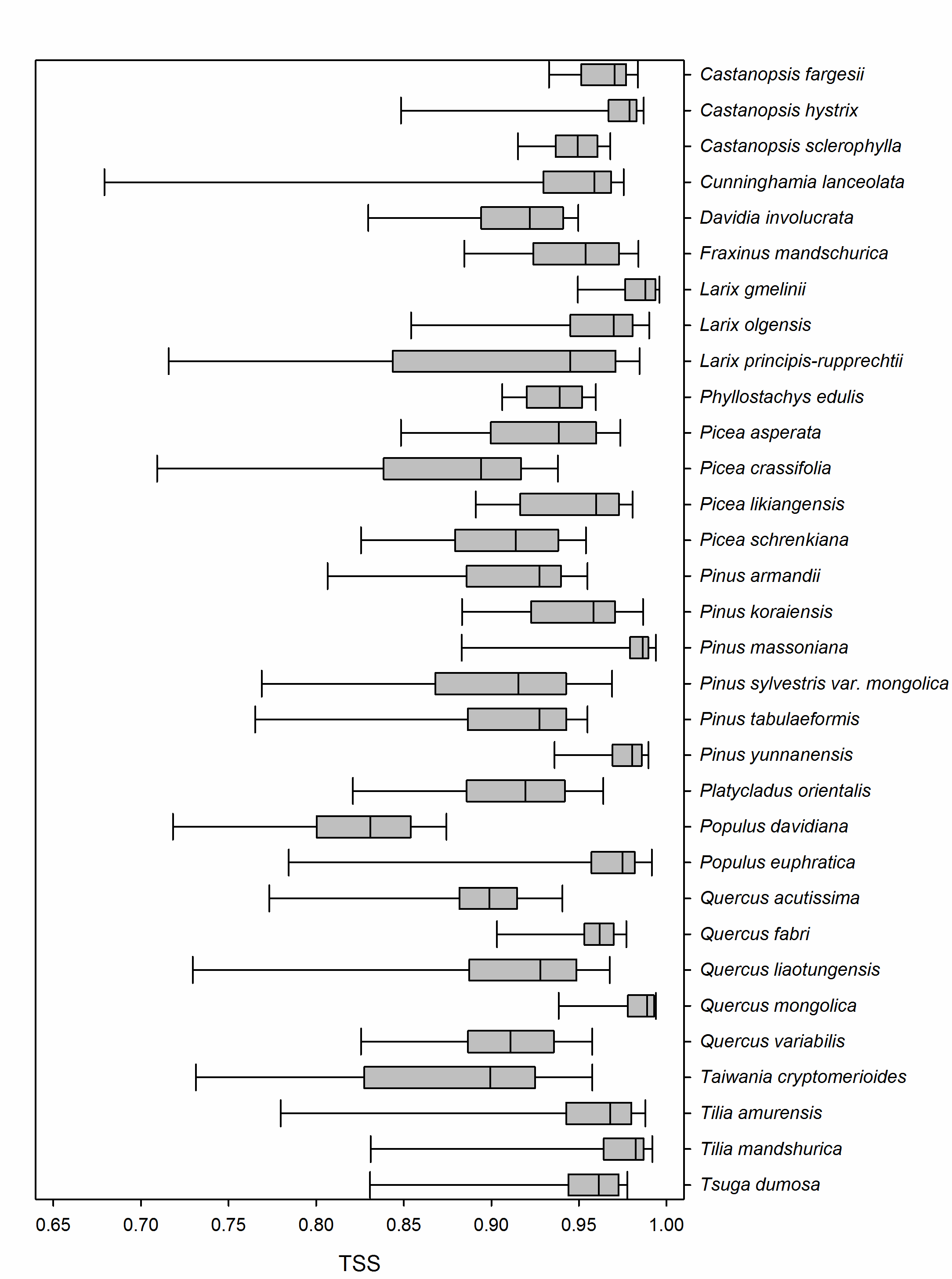

Supplement: S1 Fig — (DOC) [file pone.0120056.s005.doc]
