# Supplementary material for: Consensus Forecasting of Species Distributions: The Effects of Niche Model Performance and Niche Properties
Source: PLoS One. 2015 Mar 18;10(3):e0120056. doi: 10.1371/journal.pone.0120056 (PMC4364626; doi:10.1371/journal.pone.0120056)
Supplement: S2 Fig — (DOC) [file pone.0120056.s006.doc]

# Figure S2 Overlap maps for current and future distribution as predicted using all three consensual approaches.

**Legend**

| 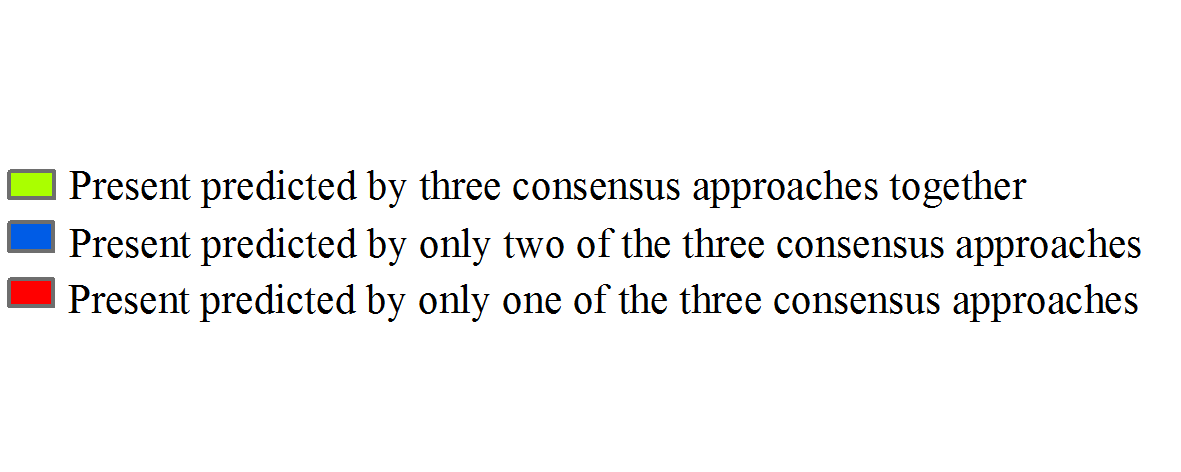 | Predicted to be present by all three consensus approaches |
| --- | --- |
| 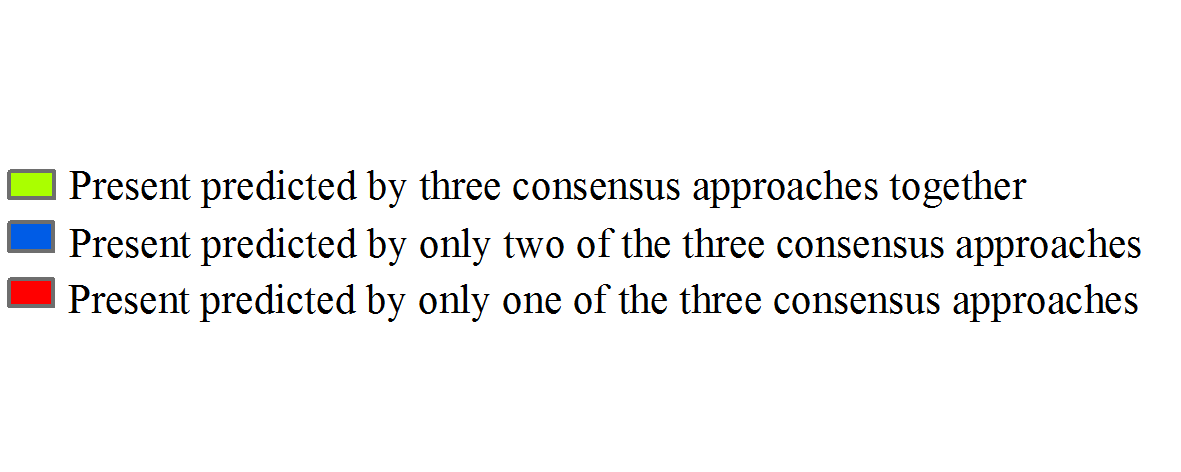 | Predicted to be present by only two of the three consensus approaches |
| 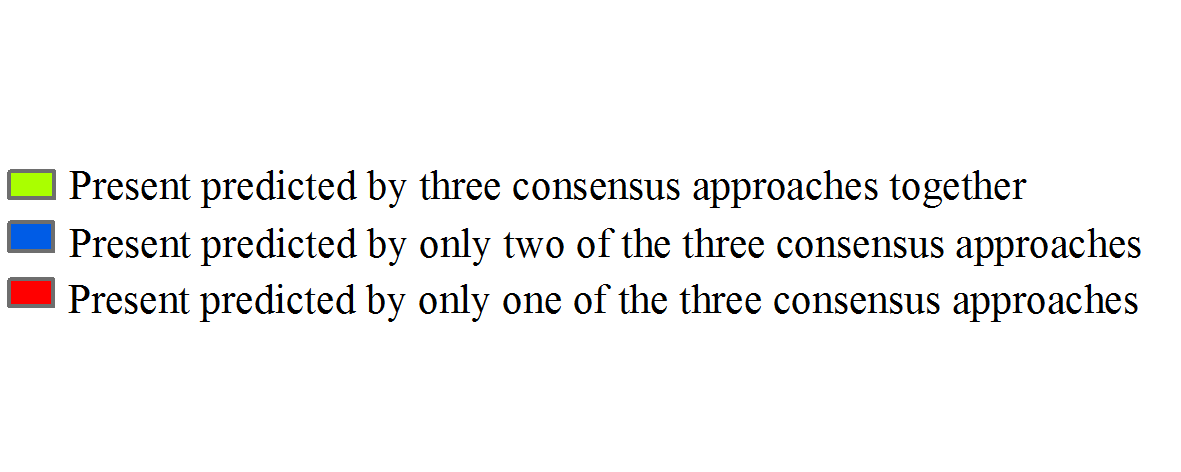 | Predicted to be present by only one of the three consensus approaches |

|  | *Castanopsis fargesii* | *Castanopsis hystrix* |
| --- | --- | --- |
| Baseline (1961–1990) | 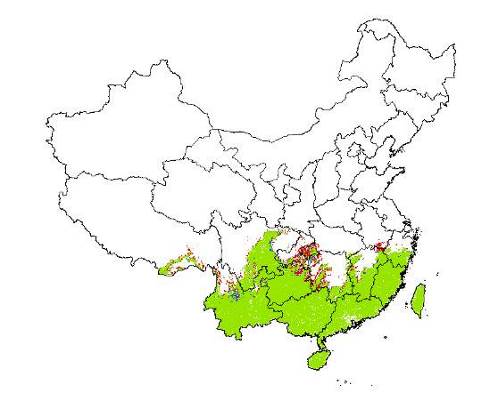 | 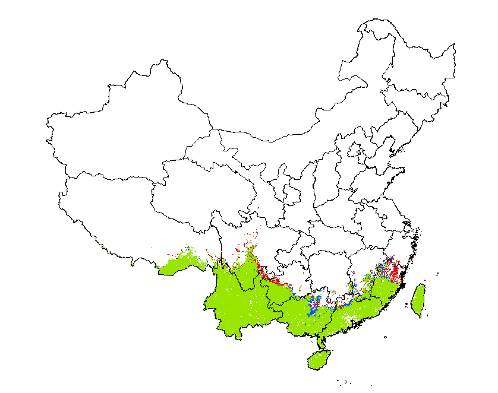 |
| 2020s (2010–2039) | 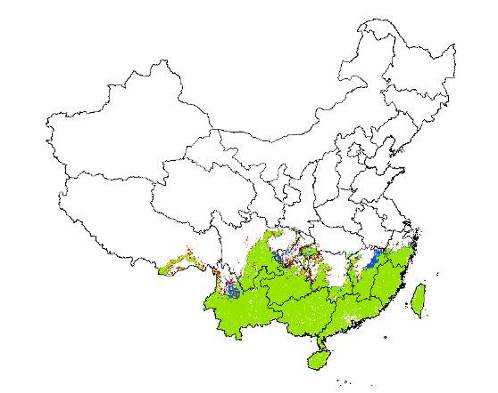 | 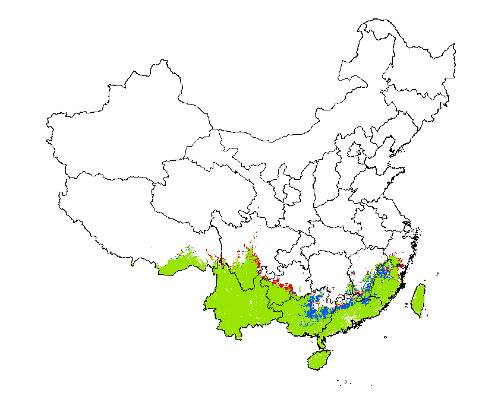 |
| 2050s (2040–2069) | 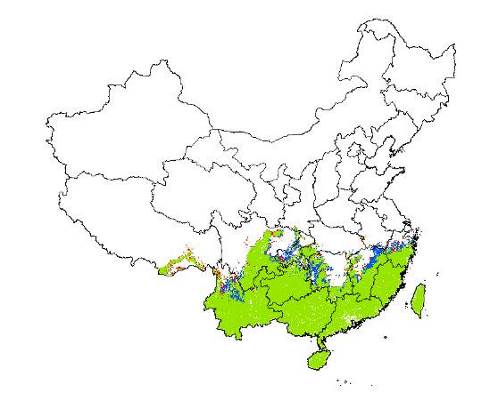 | 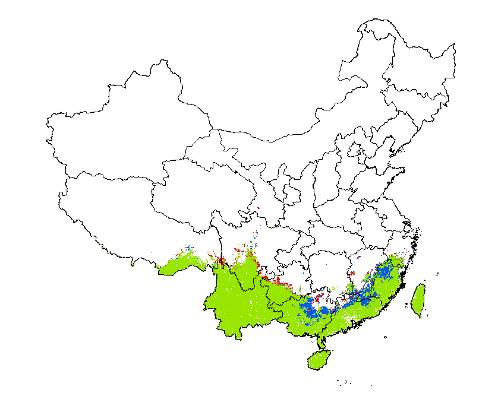 |
| 2080s (2070–2099) | 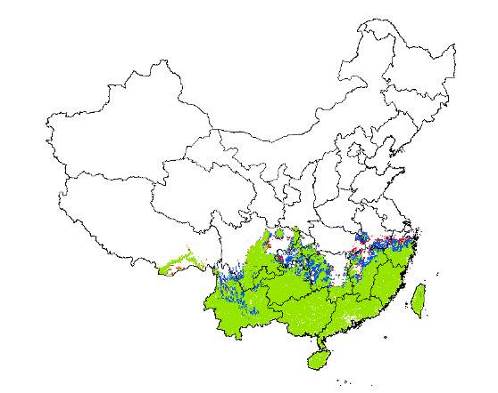 | 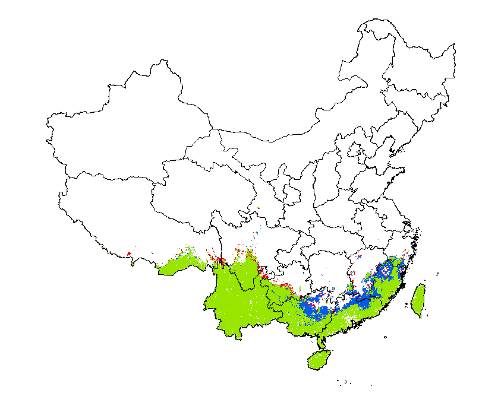 |

|  | *Castanopsis sclerophylla* | *Cunninghamia lanceolata* |
| --- | --- | --- |
| Baseline (1961–1990) | 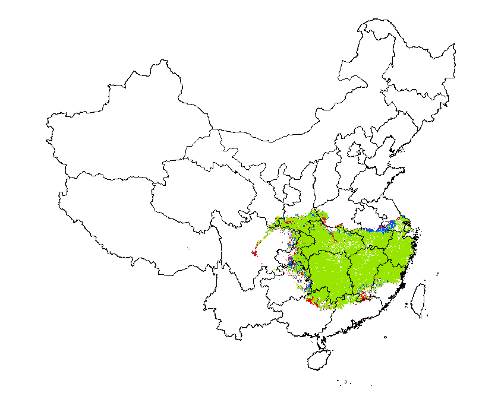 | 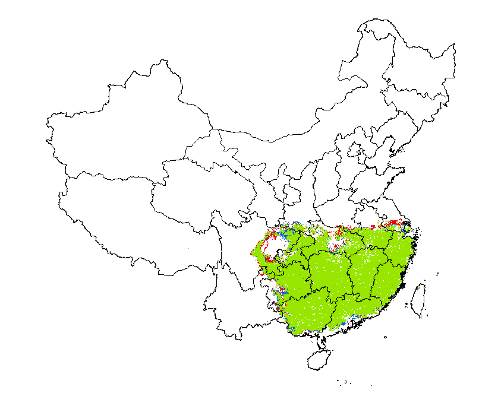 |
| 2020s (2010–2039) | 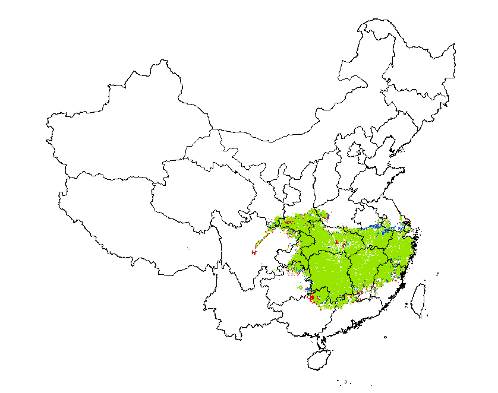 | 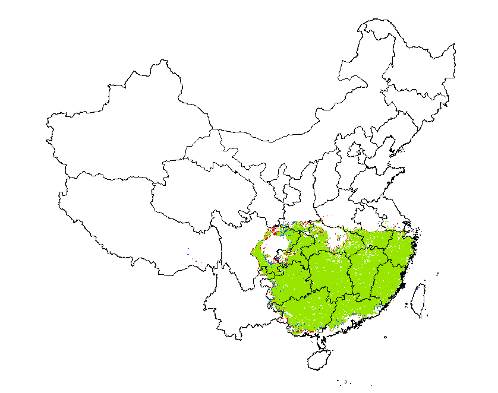 |
| 2050s (2040–2069) | 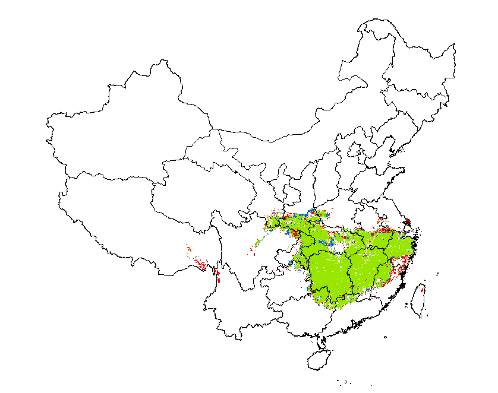 | 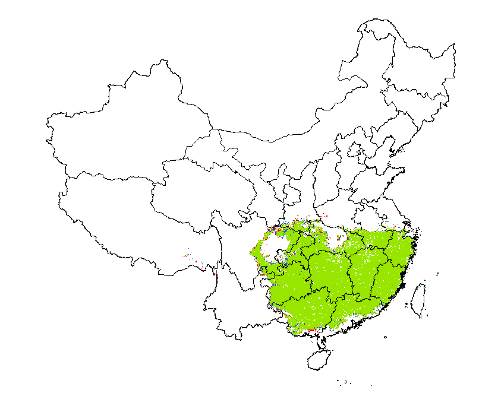 |
| 2080s (2070–2099) | 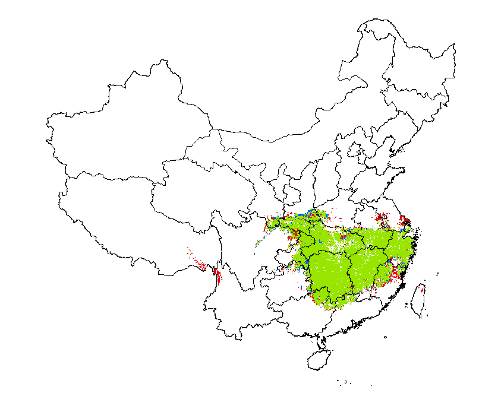 | 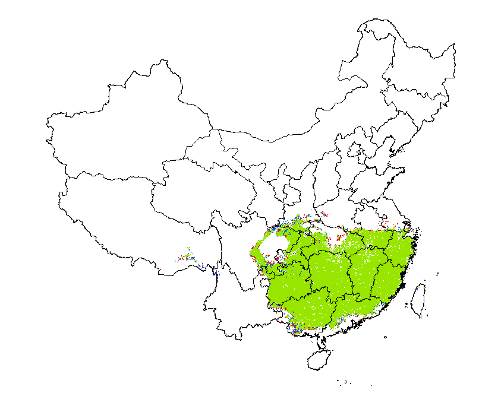 |

|  | *Davidia involucrata* | *Fraxinus mandschurica* |
| --- | --- | --- |
| Baseline (1961–1990) | 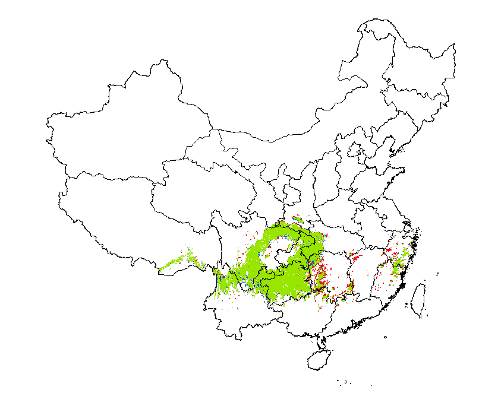 | 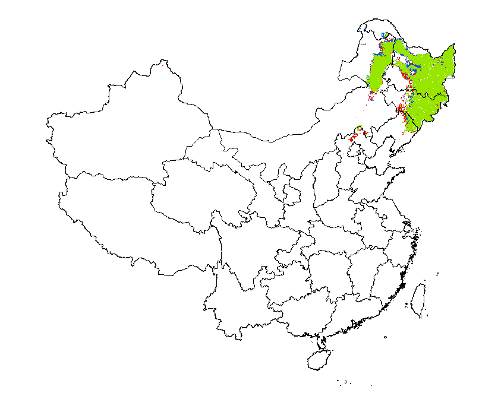 |
| 2020s (2010–2039) | 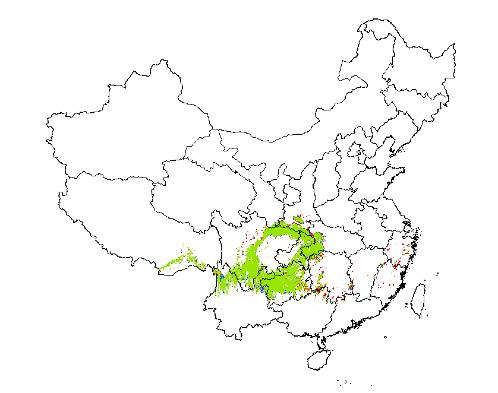 | 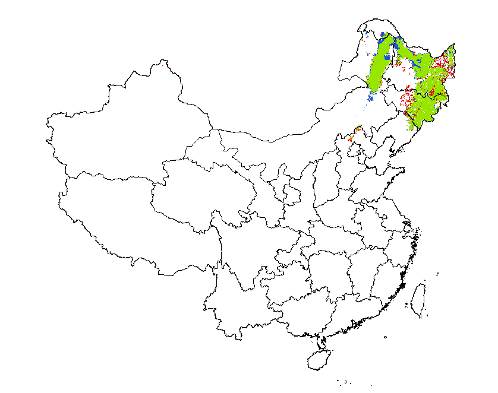 |
| 2050s (2040–2069) | 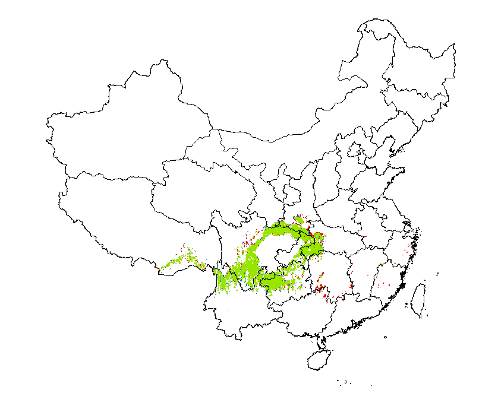 | 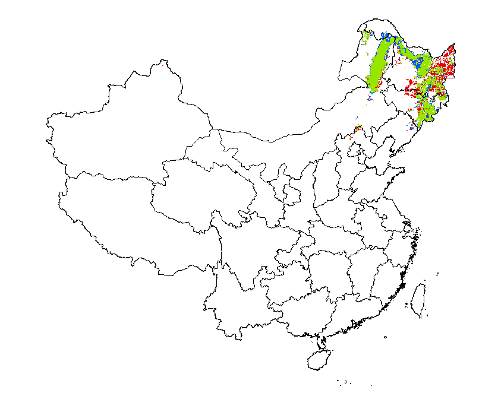 |
| 2080s (2070–2099) | 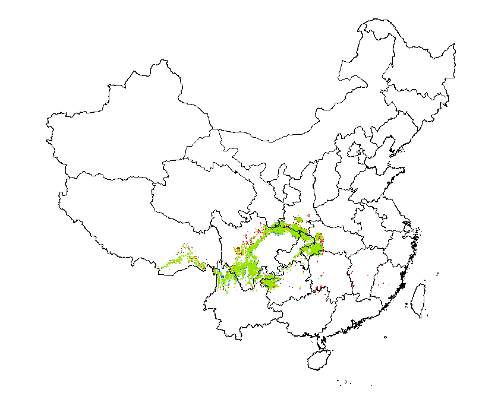 | 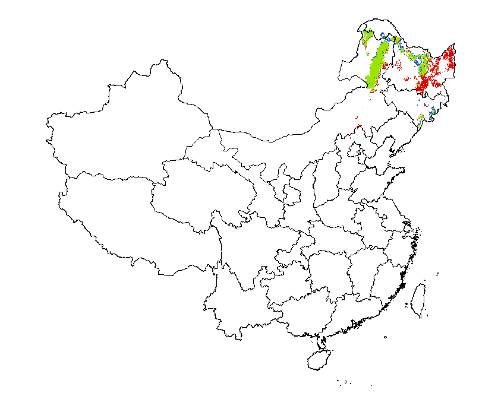 |

|  | *Larix gmelinii* | *Larix olgensis* |
| --- | --- | --- |
| Baseline (1961–1990) | 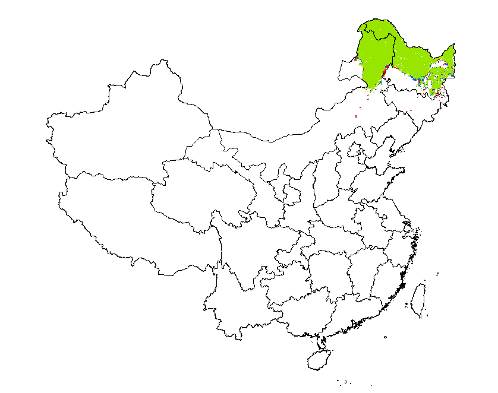 | 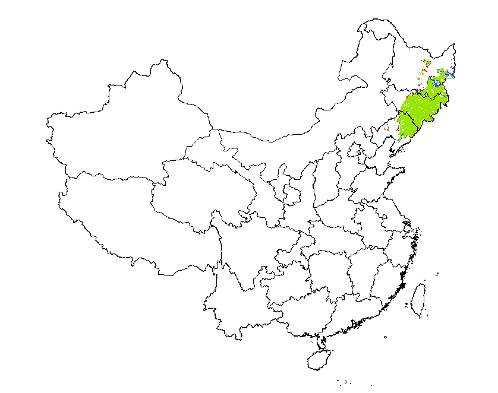 |
| 2020s (2010–2039) | 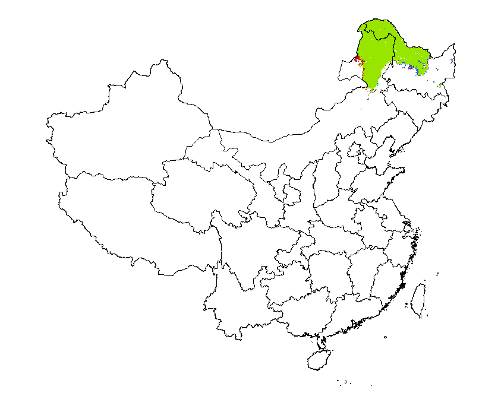 | 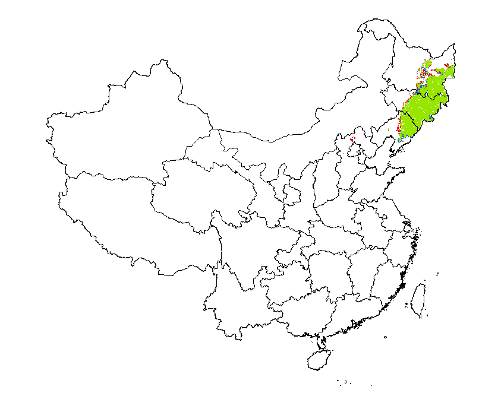 |
| 2050s (2040–2069) | 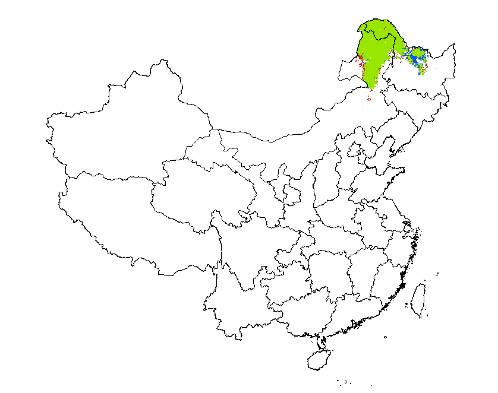 | 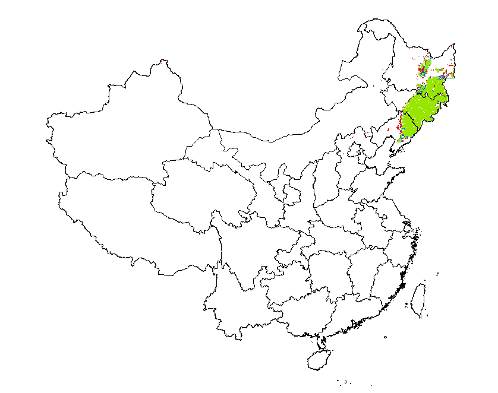 |
| 2080s (2070–2099) | 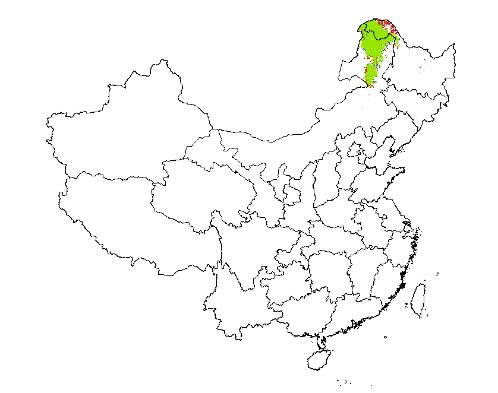 | 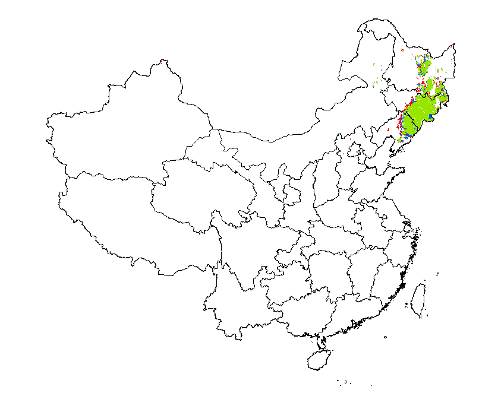 |

|  | *Larix principis–rupprechtii* | *Phyllostachys edulis* |
| --- | --- | --- |
| Baseline (1961–1990) | 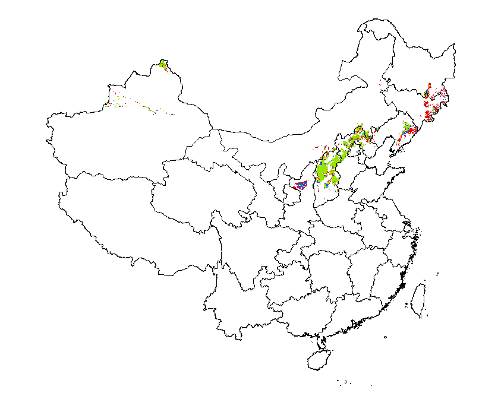 | 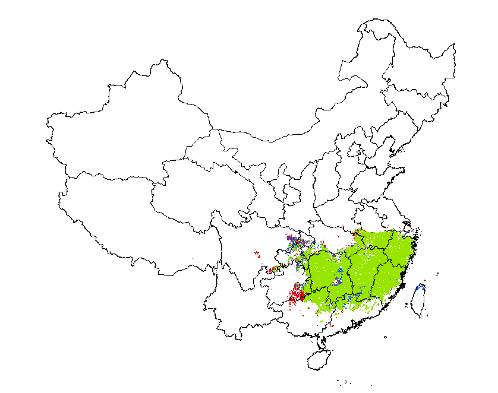 |
| 2020s (2010–2039) | 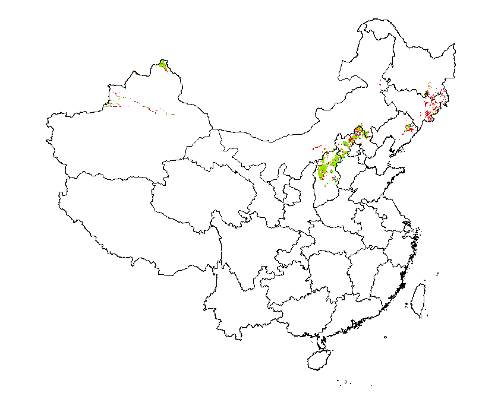 | 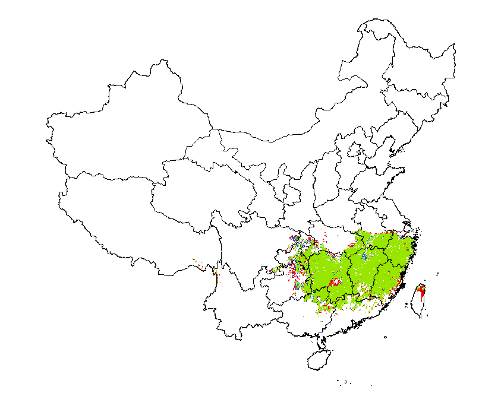 |
| 2050s (2040–2069) | 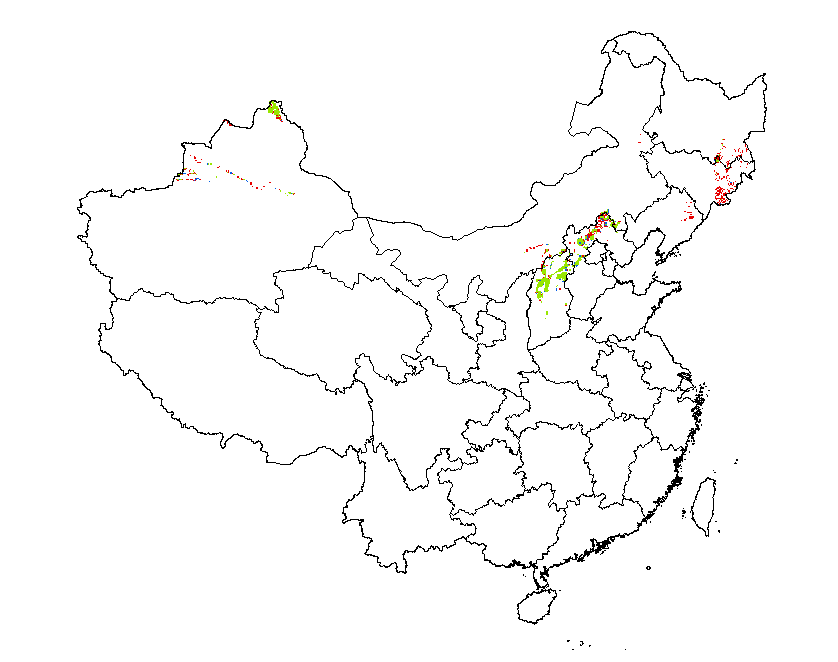 | 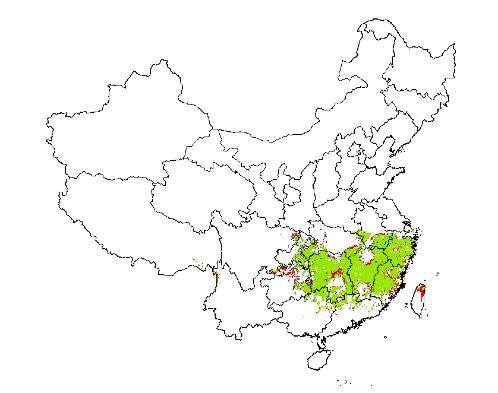 |
| 2080s (2070–2099) | 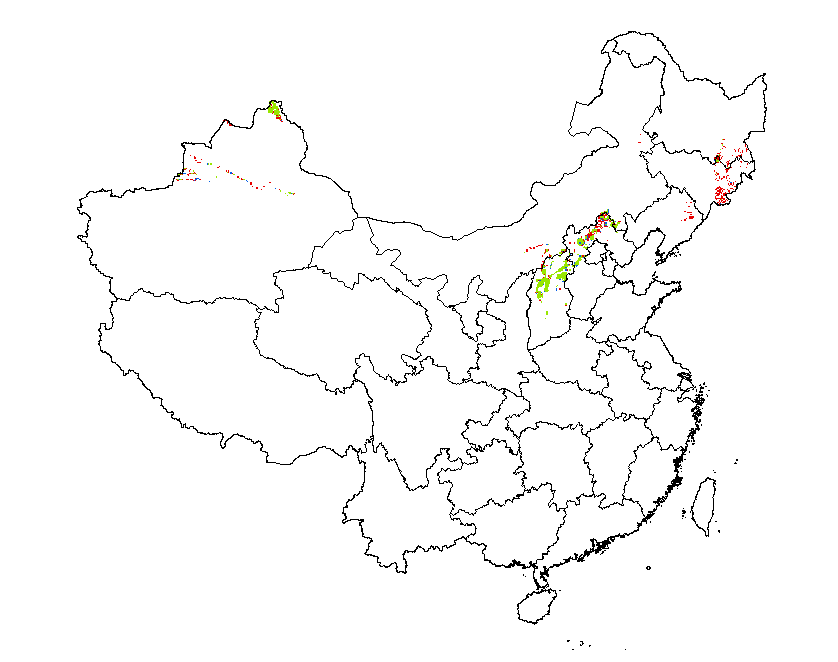 | 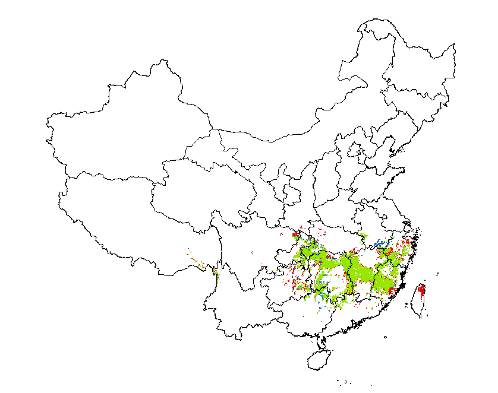 |

|  | *Picea asperata* | *Picea crassifolia* |
| --- | --- | --- |
| Baseline (1961–1990) | 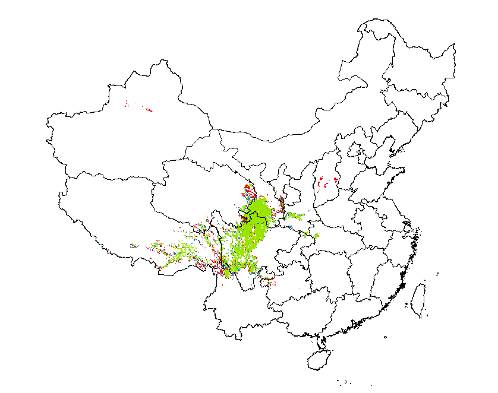 | 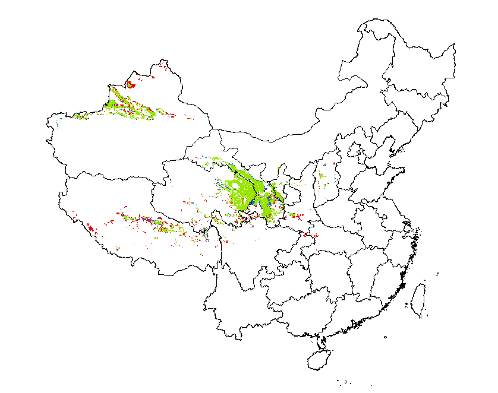 |
| 2020s (2010–2039) | 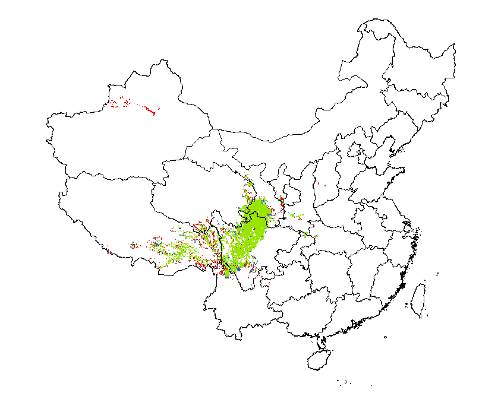 | 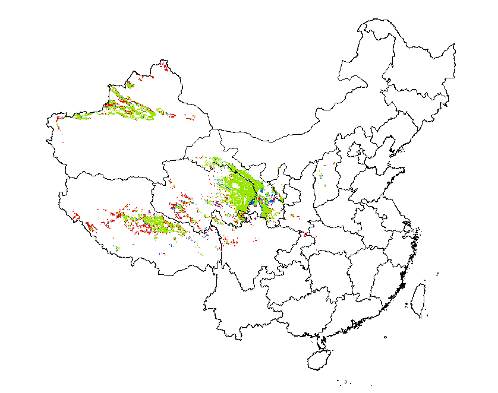 |
| 2050s (2040–2069) | 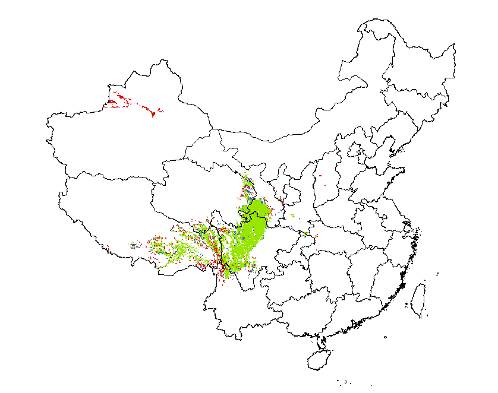 | 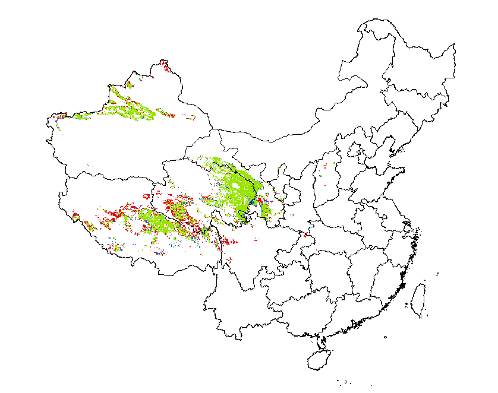 |
| 2080s (2070–2099) | 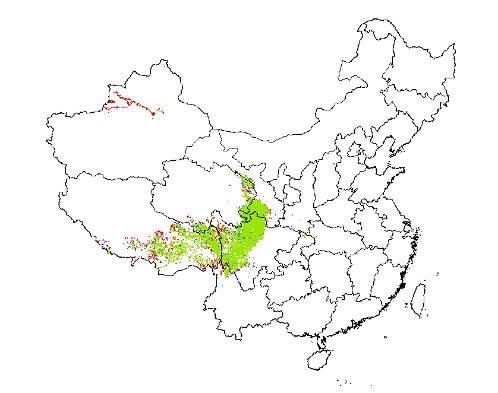 | 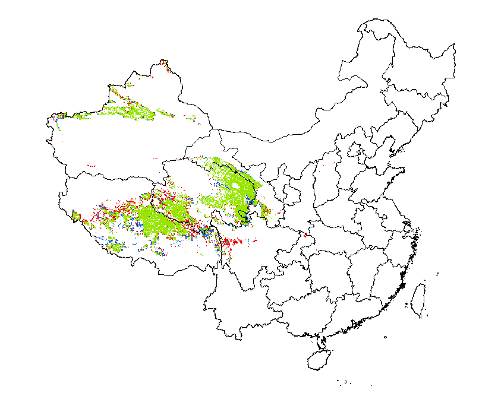 |

|  | *Picea likiangensis* | *Picea schrenkiana* |
| --- | --- | --- |
| Baseline (1961–1990) | 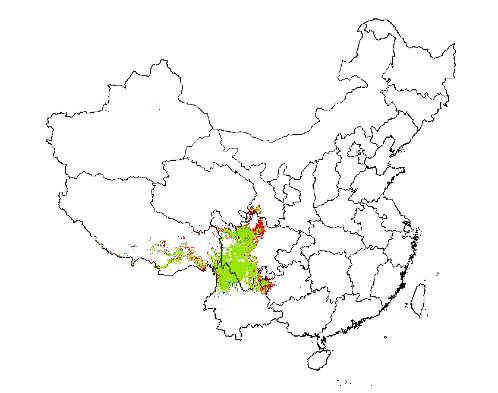 | 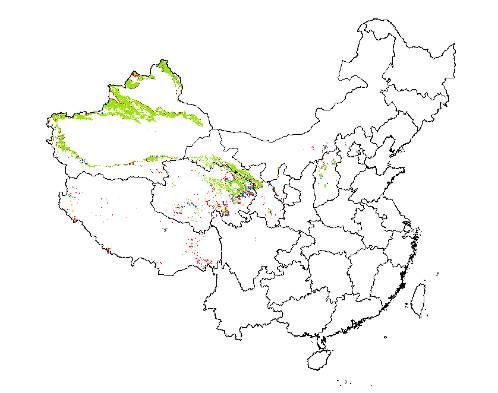 |
| 2020s (2010–2039) | 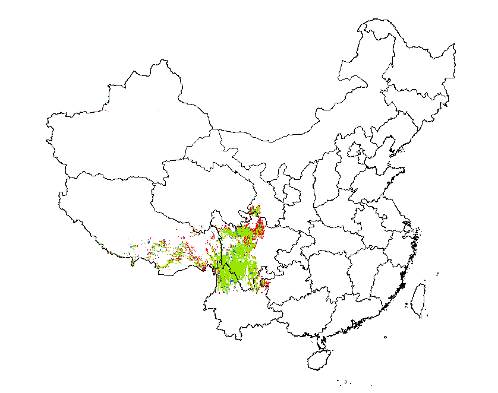 | 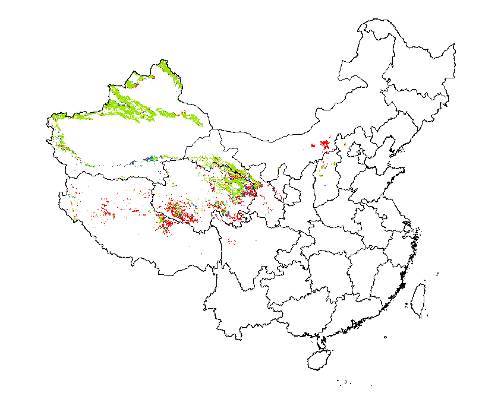 |
| 2050s (2040–2069) | 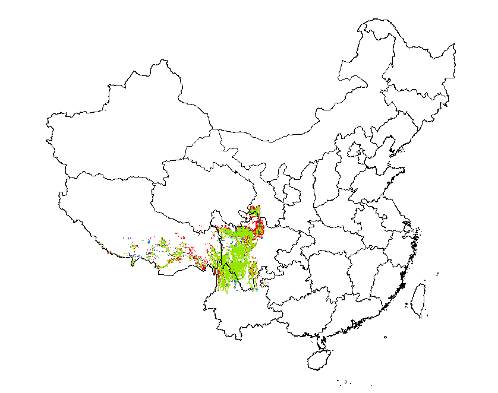 | 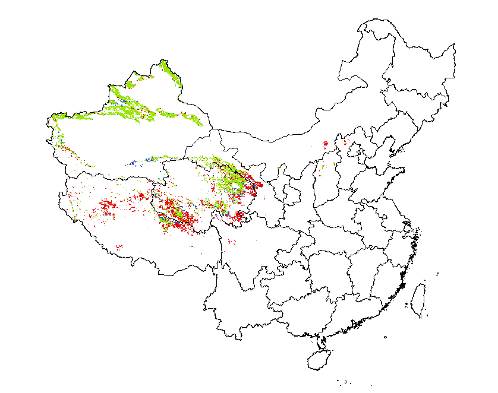 |
| 2080s (2070–2099) | 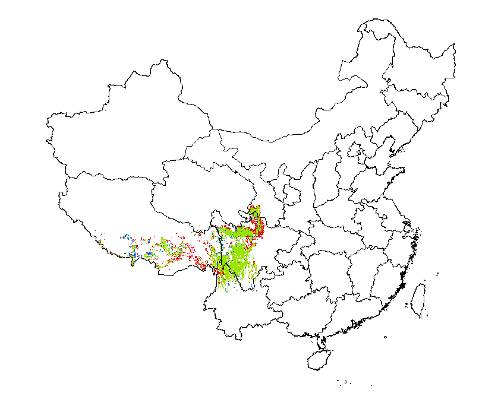 | 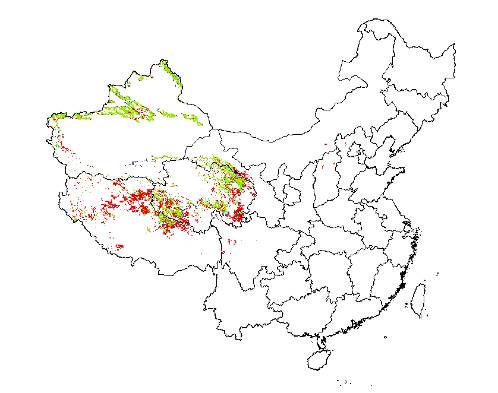 |

|  | *Pinus armandii* | *Pinus koraiensis* |
| --- | --- | --- |
| Baseline (1961–1990) | 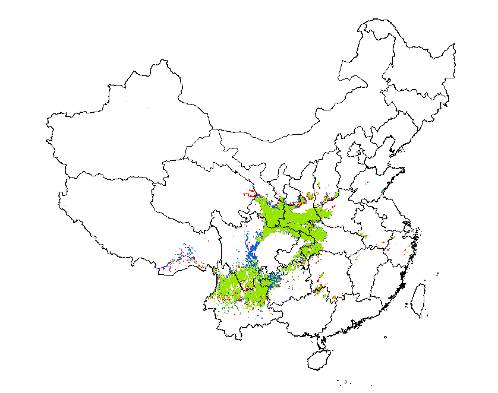 | 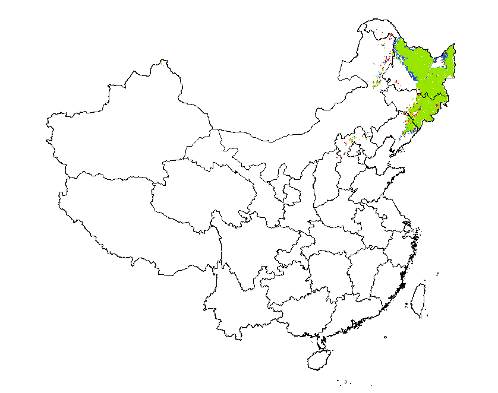 |
| 2020s (2010–2039) | 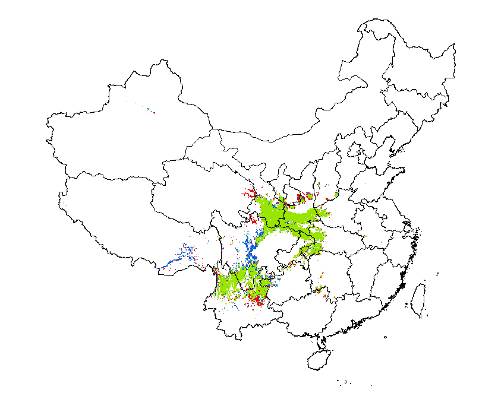 | 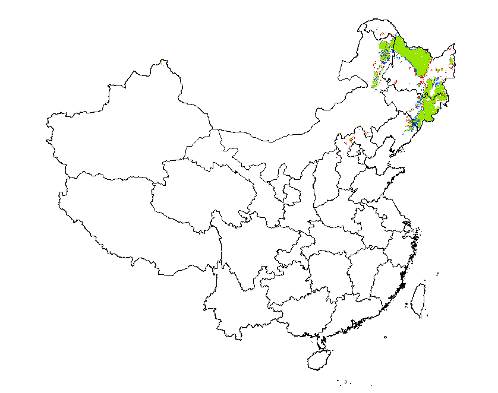 |
| 2050s (2040–2069) | 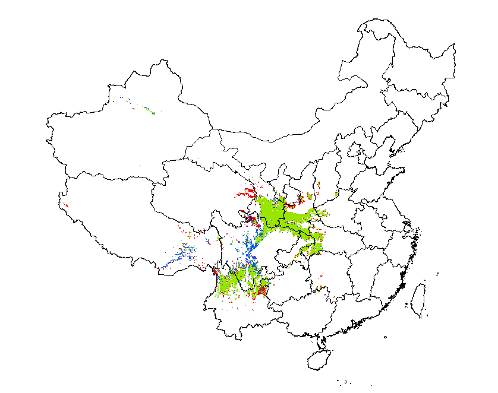 | 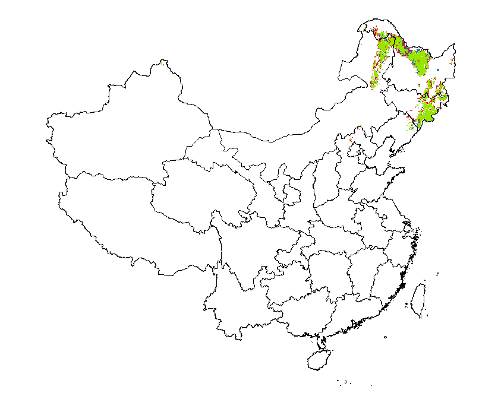 |
| 2080s (2070–2099) | 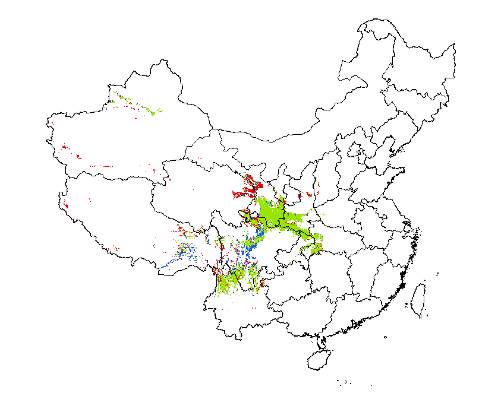 | 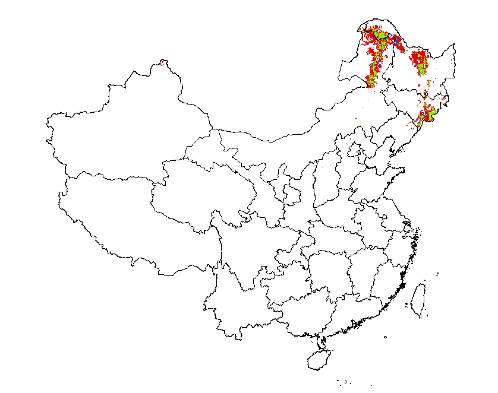 |

|  | *Pinus massoniana* | *Pinus sylvestris* var*. mongolica* |
| --- | --- | --- |
| Baseline (1961–1990) | 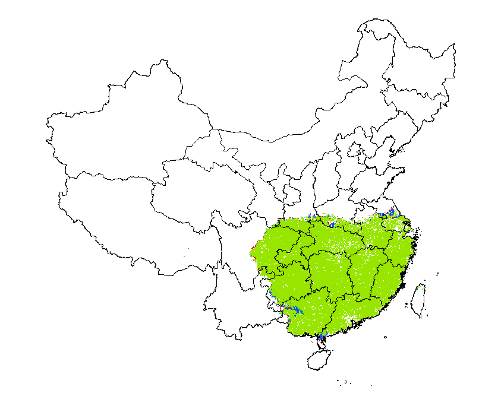 | 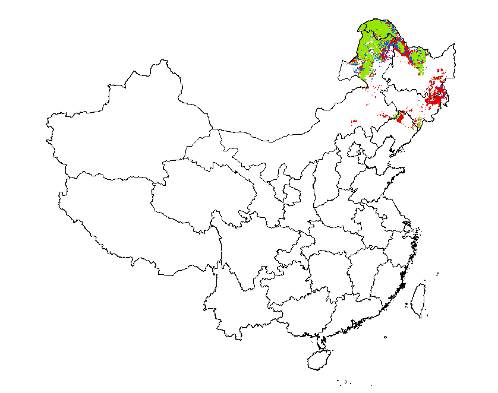 |
| 2020s (2010–2039) | 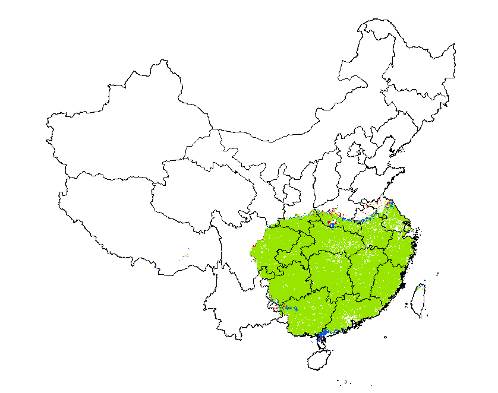 | 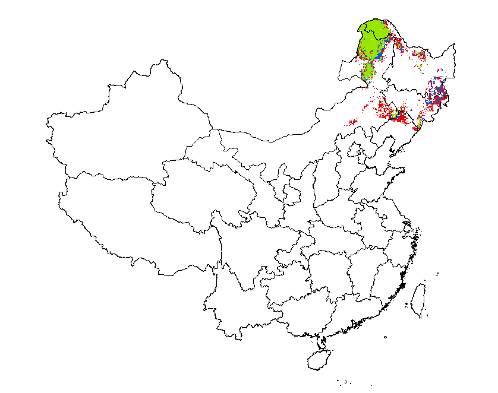 |
| 2050s (2040–2069) | 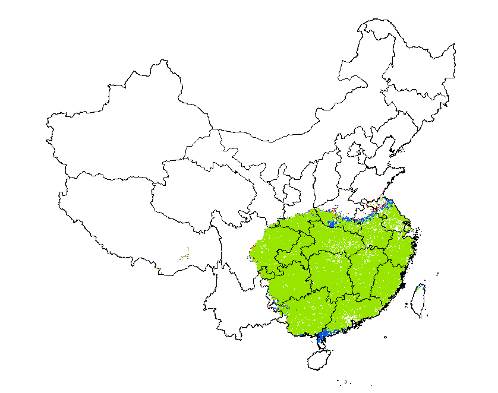 | 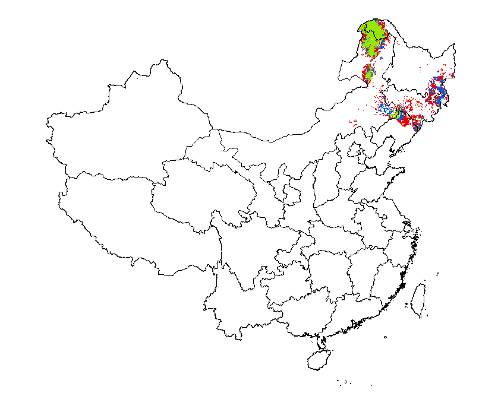 |
| 2080s (2070–2099) | 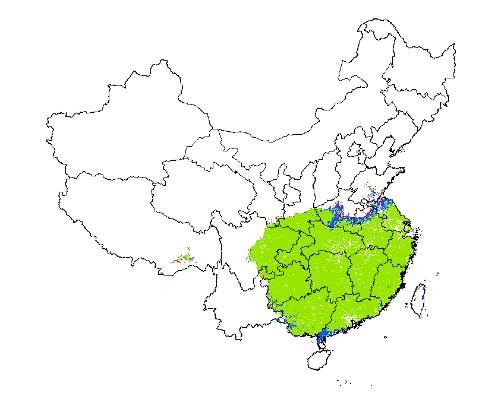 | 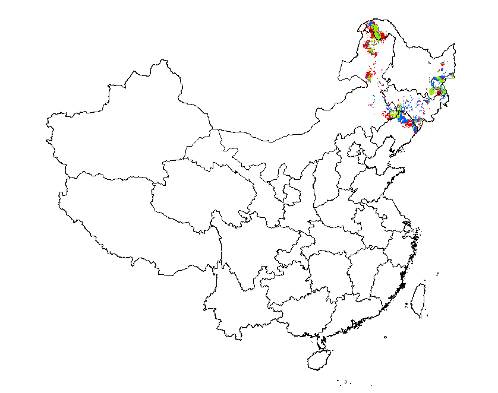 |

|  | *Pinus tabulaeformis* | *Pinus yunnanensis* |
| --- | --- | --- |
| Baseline (1961–1990) | 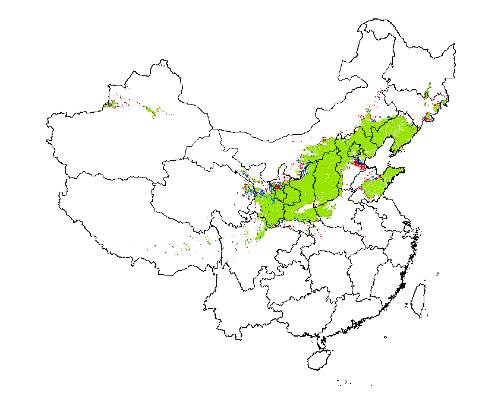 | 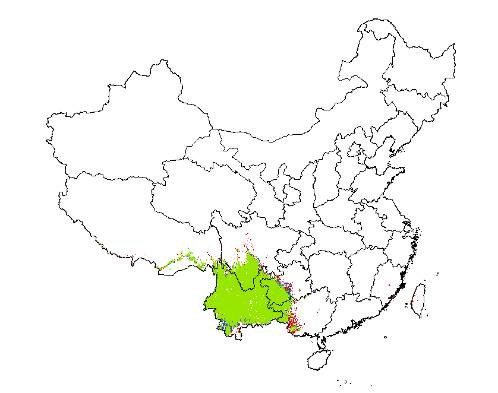 |
| 2020s (2010–2039) | 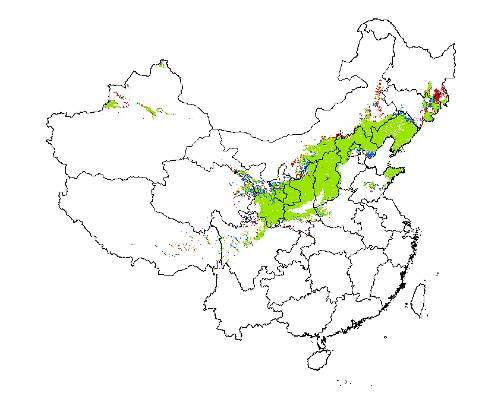 | 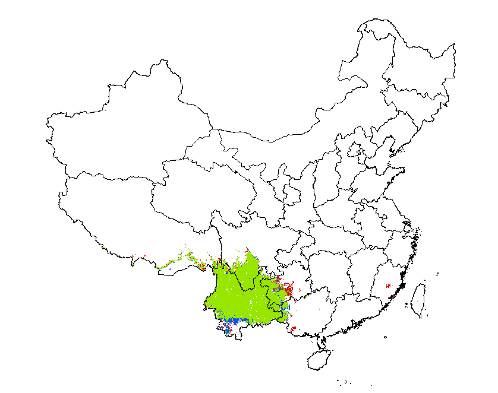 |
| 2050s (2040–2069) | 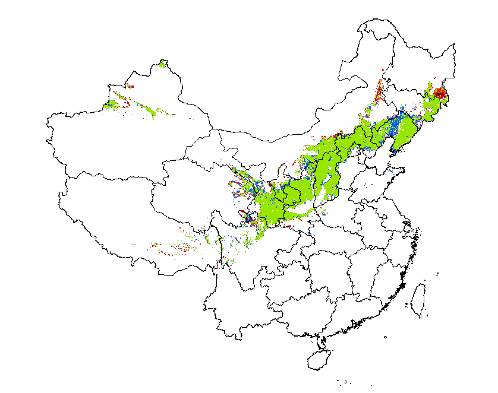 | 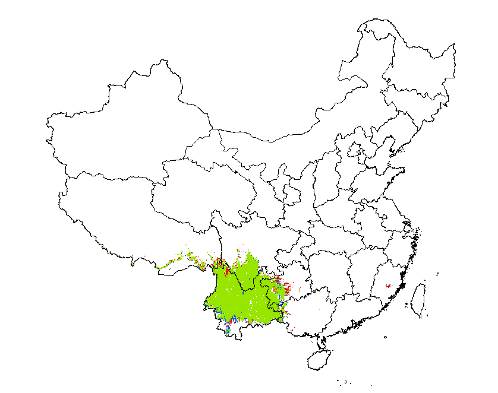 |
| 2080s (2070–2099) | 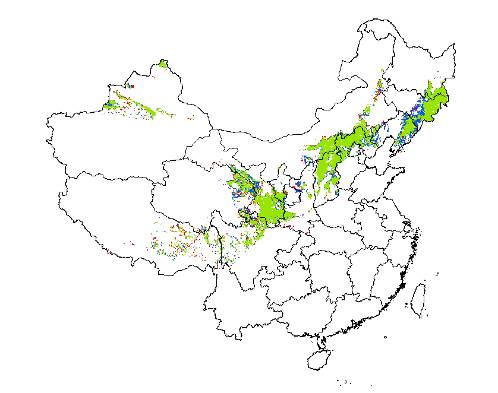 | 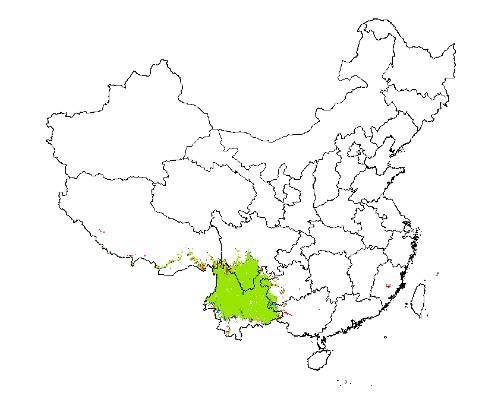 |

|  | *Platycladus orientalis* | *Populus davidiana* |
| --- | --- | --- |
| Baseline (1961–1990) | 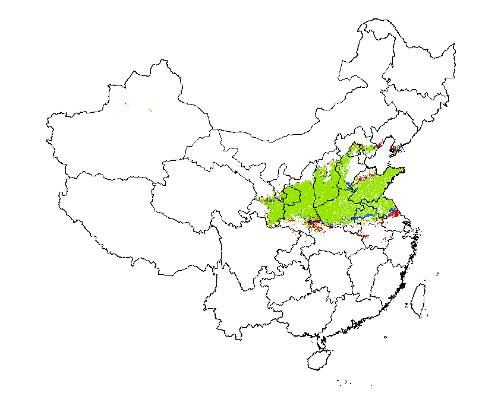 | 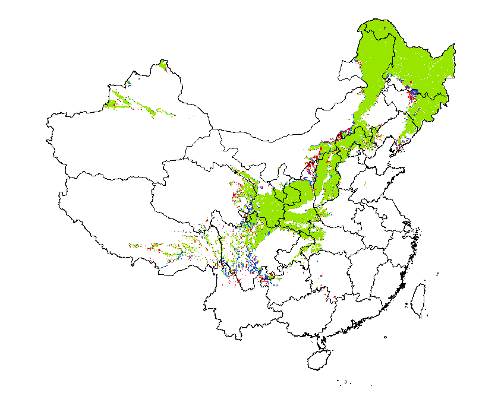 |
| 2020s (2010–2039) | 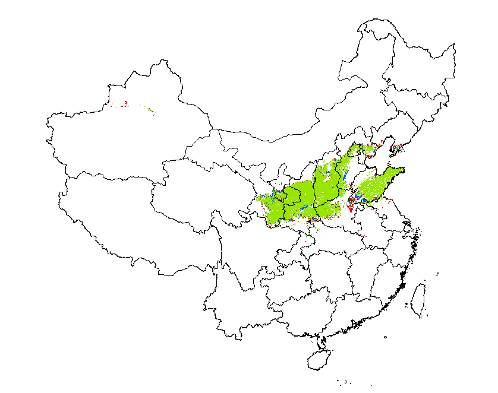 | 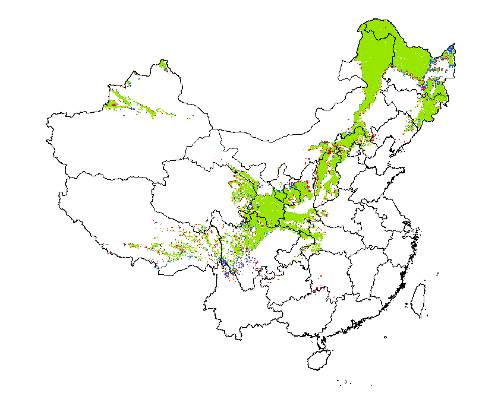 |
| 2050s (2040–2069) | 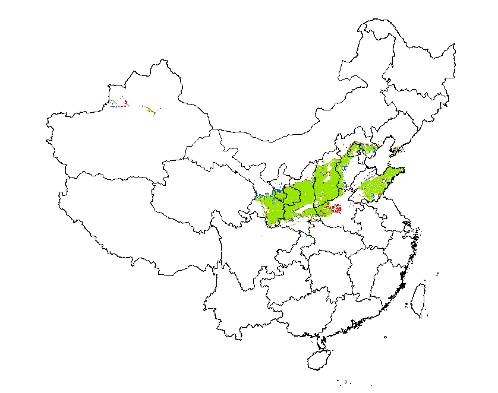 | 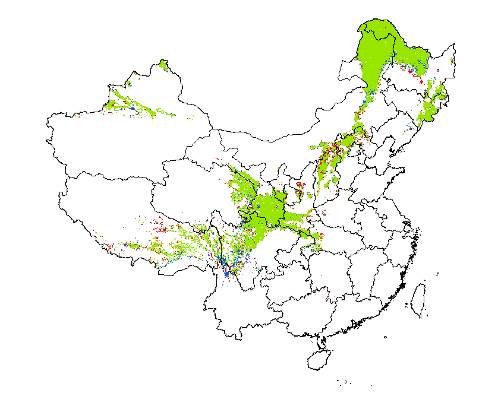 |
| 2080s (2070–2099) | 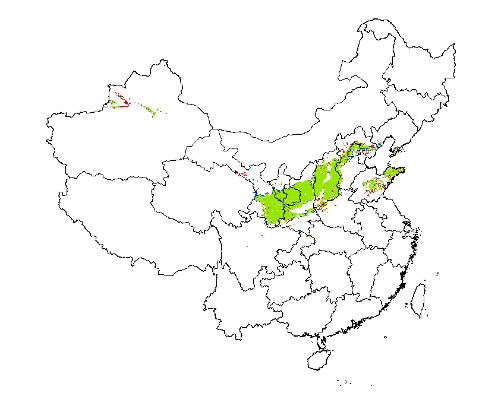 | 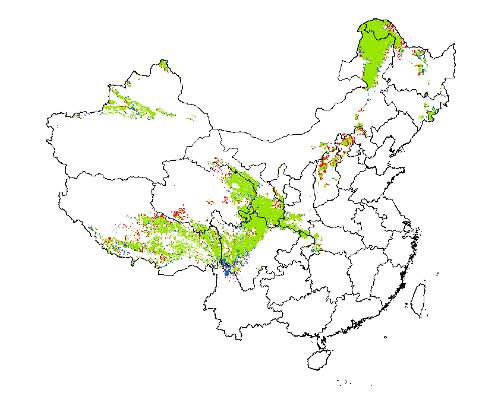 |

|  | *Populus euphratica* | *Quercus acutissima* |
| --- | --- | --- |
| Baseline (1961–1990) | 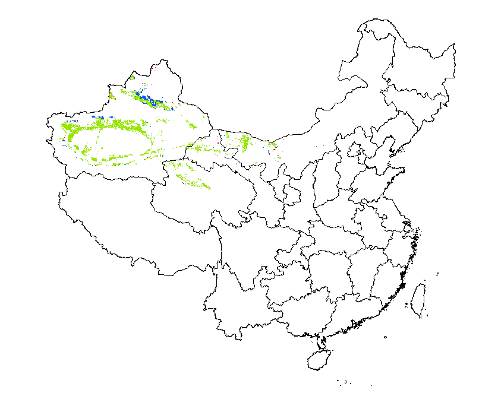 | 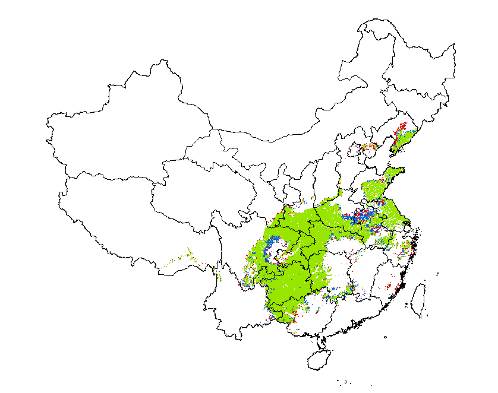 |
| 2020s (2010–2039) | 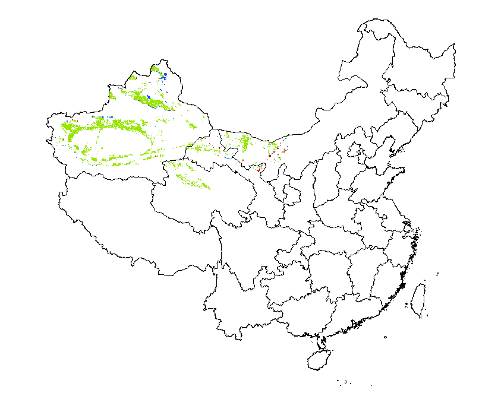 | 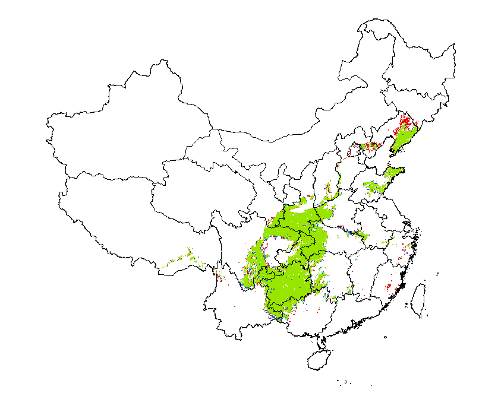 |
| 2050s (2040–2069) | 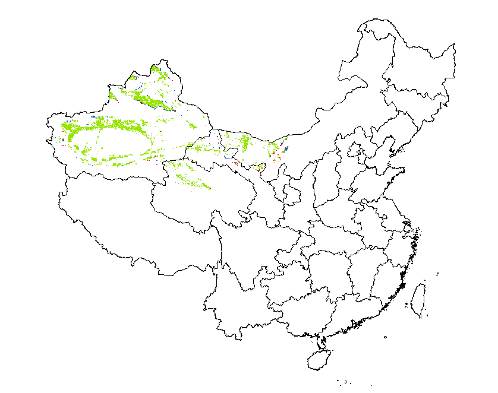 | 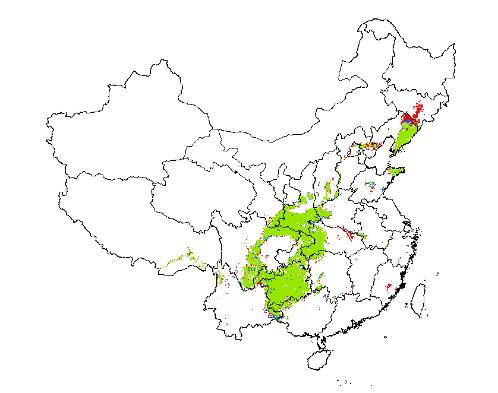 |
| 2080s (2070–2099) | 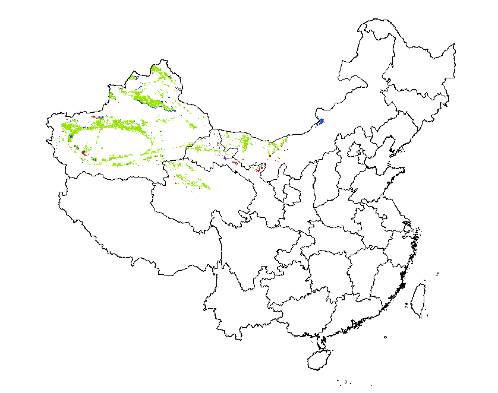 | 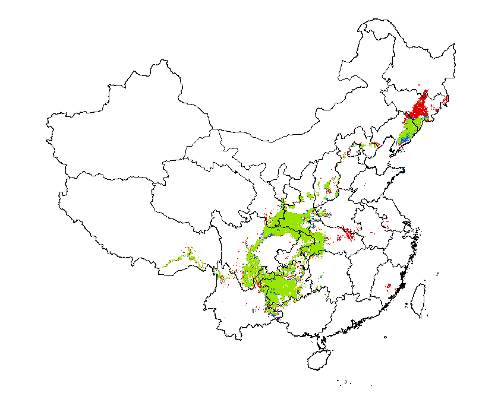 |

|  | *Quercus fabri* | *Quercus liaotungensis* |
| --- | --- | --- |
| Baseline (1961–1990) | 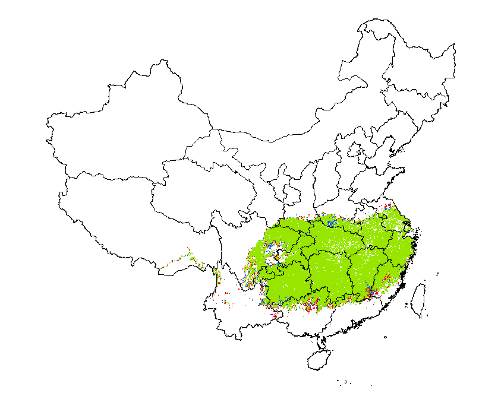 |  |
| 2020s (2010–2039) |  |  |
| 2050s (2040–2069) |  |  |
| 2080s (2070–2099) |  |  |

|  | *Quercus mongolica* | *Quercus variabilis* |
| --- | --- | --- |
| Baseline (1961–1990) |  |  |
| 2020s (2010–2039) |  |  |
| 2050s (2040–2069) |  |  |
| 2080s (2070–2099) |  |  |

|  | *Taiwania cryptomerioides* | *Tilia amurensis* |
| --- | --- | --- |
| Baseline (1961–1990) |  |  |
| 2020s (2010–2039) |  |  |
| 2050s (2040–2069) |  |  |
| 2080s (2070–2099) |  |  |

|  | *Tilia mandshurica* | *Tsuga dumosa* |
| --- | --- | --- |
| Baseline (1961–1990) |  |  |
| 2020s (2010–2039) |  |  |
| 2050s (2040–2069) |  |  |
| 2080s (2070–2099) |  |  |
