# Supplementary material for: Consensus Forecasting of Species Distributions: The Effects of Niche Model Performance and Niche Properties
Source: PLoS One. 2015 Mar 18;10(3):e0120056. doi: 10.1371/journal.pone.0120056 (PMC4364626; doi:10.1371/journal.pone.0120056)
Supplement: S1 Appendix — (DOC) [file pone.0120056.s007.doc]

# Appendix S1. Description of the software ClimateChina.

ClimateChina (Ver4.4) application was jointly developed by University of British Columbia and Chinese Academy of Forestry using the same methodology as ClimateBC (Zhang et al., 2011; Wang et al., 2006). The program extracts and downscales PRISM 1961–1990 monthly normal data (2.5 × 2.5 arcmin) to scale-free seamless climate data (Daly et al., 2002). It calculates seasonal and annual climate variables for specific locations based on latitude, longitude, and elevation (optional). Downscaling of PRISM monthly data is achieved through a combination of bilinear interpolation and elevation adjustment. Calculations of climate variables and estimation of derived climate variables are described in a report by Wang et al. (2006). The program uses scale-free data as baseline and monthly anomaly data as a baseline of individual years to calculate historical monthly, seasonal and annual climate variables for individual years and periods from 1901–2002 (CRU 2.1, Mitchell & Jones, 2005). This program also downscales and integrates future climate datasets generated using various GCMs. The spatial coverage of this software extends over all of China.

**References**

Daly, C., Gibson, W.P., Taylor, G.H., Johnson, G.L. and Pasteris, P., 2002. A knowledge-based approach to the statistical mapping of climate. climate Research, 22:99–113.

Mitchell, T.D. and Jones, P.D., 2005. An improved method of constructing a database of monthly climate observations and associated high-resolution grids. International Journal of Climatology, 25:693–712.

Wang, T., Hamann, A., Spittlehouse, D.L. and Aitken, S.N., 2006. Development of scale-free climate data for western Canada for use in resource management. International Journal of Climatology, 26:383–397.

Zhang, L., S. Liu, P. Sun, and T. Wang. 2011. Comparative evaluation of multiple models of the effects of climate change on the potential distribution of *Pinus massoniana*. Chinese Journal of Plant Ecology 35:1091–1105. (in Chinese with English abstract).
